# Crystal structure of bis­[(phenyl­methanamine-κ*N*)(phthalocyaninato-κ^4^ *N*)zinc] phenyl­methan­amine tris­olvate

**DOI:** 10.1107/S2056989015014280

**Published:** 2015-08-12

**Authors:** Norzianah Shamsudin, Ai Ling Tan, Franz L. Wimmer, David J. Young, Edward R. T. Tiekink

**Affiliations:** a Faculty of Science, Universiti Brunei Darussalam, Jalan Tungku Link BE 1410, Negara Brunei Darussalam; b Faculty of Science, Health, Education and Engineering, University of the Sunshine Coast, Maroochydore DC, Queensland 4558, Australia; c Department of Chemistry, University of Malaya, 50603 Kuala Lumpur, Malaysia

**Keywords:** crystal structure, zinc phthalocyaninato complex, co-crystal, hydrogen bonding, conformation

## Abstract

The asymmetric unit of the title compound, 2[Zn(C_32_H_16_N_8_)(C_7_H_9_N)]·3C_7_H_9_N, comprises two independent complex mol­ecules and three benzyl­amine solvent mol­ecules. Each complex mol­ecule features a penta­coordinated Zn^2+^ ion within a square-pyramidal geometry, whereby the N_5_ donor set is defined by four atoms of the phthalocyaninate dianion (PC) and an N-bound benzyl­amine mol­ecule; it is the relative orientations of the latter that differentiate between the independent complex mol­ecules. The uncoordinated benzyl­amine mol­ecules display different conformations in the structure, with *syn*-C_ar_—C_ar_—C_m_—N (ar = aromatic, m = methyl­ene) torsion angles spanning the range −28.7 (10) to 35.1 (14)°. In the crystal, N—H⋯N and N—H⋯π inter­actions lead to supra­molecular layers in the *ab* plane. The layers have a zigzag topology, have the coordinating and non-coordinating benzyl­amine mol­ecules directed to the inside, and present the essentially flat PC resides to the outside. This arrangement enables adjacent layers to associate *via* π–π inter­actions [inter-centroid distance between pyrrolyl and fused-benzene rings = 3.593 (2) Å] so that a three-dimensional architecture is formed.

## Abstract 2

A penta­coordinated Zn^2+^ ion is found in each independent complex mol­ecule of the title compound; the asymmetric unit is completed by three conformationally flexible non-coordinating benzyl­amine mol­ecules. Supra­molecular layers sustained by N—H⋯N and N—H⋯π inter­actions are found in the crystal packing; these are connected by π–π contacts.

## Chemical context   

Phthalocyanines of most main group metals and semi-metals, transition metals, lanthanides and actinides are known. Recent inter­est has centered on their electronic, photoelectronic and catalytic properties for a diverse array of applications including photodynamic therapy (Bonnett, 1995 ▸), as semi-conducting materials (Yang *et al.*, 2015 ▸), as homogeneous and heterogeneous catalysts (Sorokin, 2013 ▸), as dyes for dye-sensitive solar cells (DSSC) (Ince *et al.*, 2014 ▸), in chemical sensors (Zhang *et al.*, 2015 ▸) and for optical data storage (de la Torre *et al.*, 2007 ▸). The first metal phthalocyanine identified was Fe phthalocyanine (FePC), prepared in 1928 as a by-product during the industrial production of phthalimide from the reaction of ammonia with molten phthalic anhydride in an Fe vessel (Linstead, 1934 ▸). The intense blue colour of this thermally stable material was subsequently exploited in paints and textile dyes. Another defining characteristic is the insolubility of FePC in water, common organic solvents, dilute acids and alkali. It was, however, soluble ‘in hot aniline and its homologues to give intensely green solutions which contained complex additive compounds’ (Linstead, 1934 ▸). Robertson and Woodward achieved the first complete X-ray crystallographic elucidation of a family member, NiPC, confirming the planar, tetra-iso­indole macrocyclic structure with tetracoordinated metal (Robertson & Woodward, 1937 ▸).

Zinc phthalocyanine (ZnPC) is one of the more soluble members of the transition metal phthalocyanines, although a saturated solution in NMP (*N*-methyl-2-pyrrolidone) is still less than 7 m*M* (Ghani *et al.*, 2012 ▸). This limits its wet processibility. ZnPC is known to form a weak complex with one pyridine ligand (Taube, 1974 ▸). We have an on-going inter­ested in doped TiO_2_ for use as DSSC photoanodes (Ako *et al.*, 2015 ▸) and investigated the use of solutions of ZnPC in benzyl­amine for coating TiO_2_ nanoparticles. This high-boiling primary amine proved to be a reasonable solvent for this dye. It was during the course of these studies that crystals of the title compound, (I), were isolated. The crystallographic characterization of (I) is described herein along with its comparison to related ZnPC adducts with N-donors. A discussion of the conformational variability of uncoordinated benzyl­amine is also included.
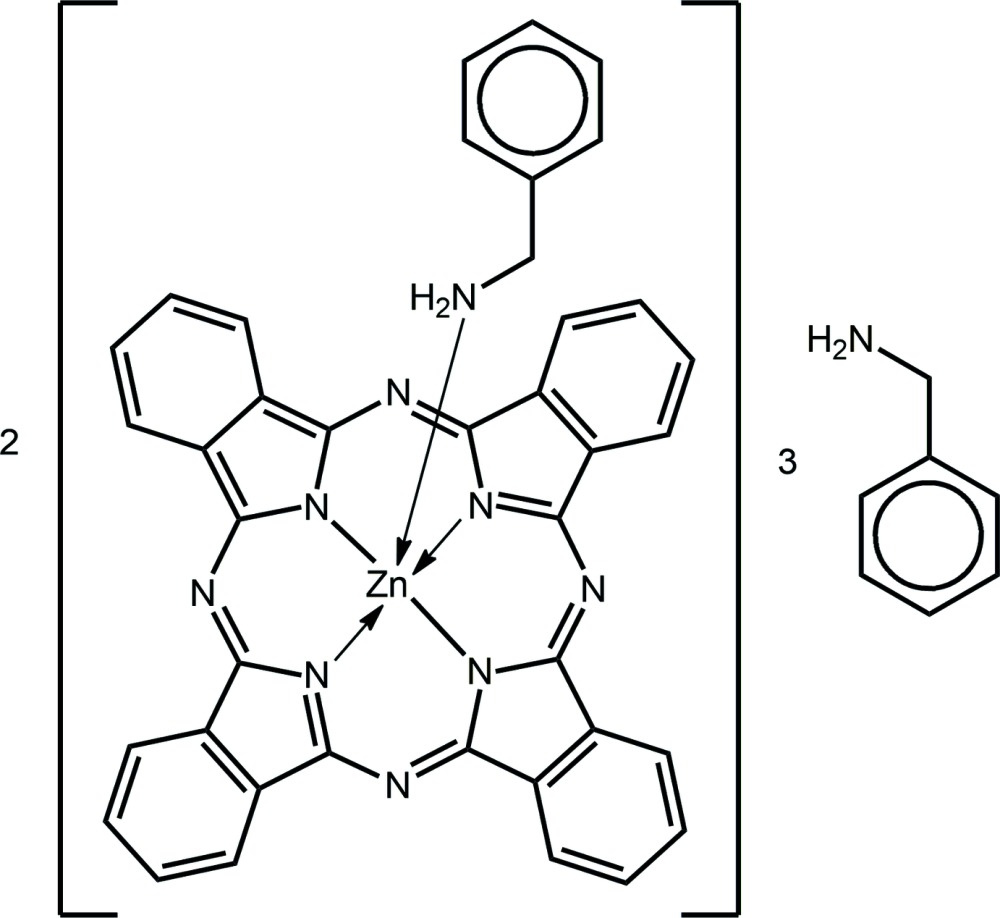


## Structural commentary   

**Figure 1:**
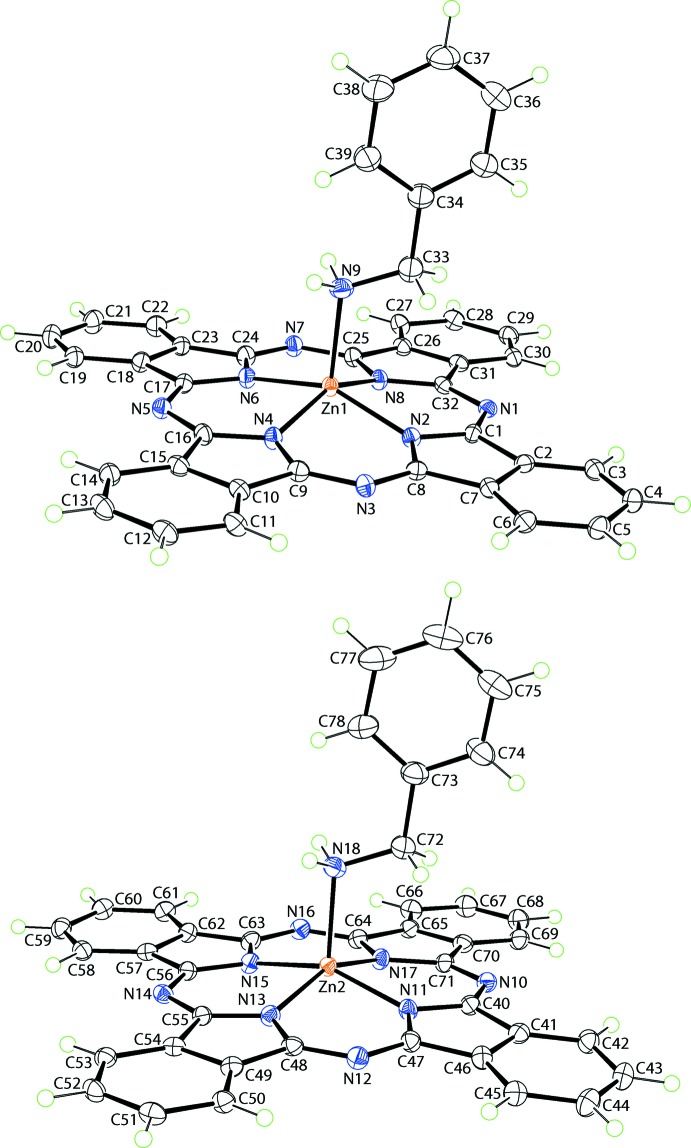
The mol­ecular structures of the two independent complex mol­ecules in (I), showing the atom-labelling scheme and displacement ellipsoids at the 50% probability level.

The asymmetric unit of (I) comprises two independent complex mol­ecules and three solvent benzyl­amine mol­ecules. Fig. 1 ▸ shows the two complex mol­ecules in which each Zn atom is coordinated by four N atoms derived from the phthalocyaninato (PC) dianion, as well as the amino-N atom from the benzyl­amine mol­ecule. The coordination of the PC dianion leads to the formation of four linked ZnNCNCN chelate rings, each of which may be described as having an envelope conformation with the Zn atom being the flap atom. An inspection of the Zn—N(PC) bond lengths collated in Table 1 ▸ shows that these span a narrow range, *i.e*. 2.025 (3) Å [Zn1—N6] to 2.045 (3) [Zn1—N4] Å, suggesting extensive delocalization of π-electron density over the PC chromophore. Further, the Zn—N(PC) bond lengths are systematically shorter than the Zn—N(amino) bonds. The N_5_ donor set defines an approximately square-pyramidal geometry with the benzyl­amino-N atoms occupying the axial position. In this description, Zn1 lies 0.4670 (16) Å above the least-squares plane defined by the four PC-N atoms (r.m.s. deviation = 0.0104 Å) in the direction of the benzyl­amino-N atom [2.570 (4) Å above the plane]; the comparable values for the Zn2-containing mol­ecule are 0.4365 (16), 0.0076 and 2.549 (4) Å, respectively. That the N_5_ donor set defines a square pyramid is qu­anti­fied by the value of τ = 0.02 for each of the Zn1- and Zn2-containing mol­ecules, which compares to the τ values of 0.0 and 1.0 for ideal square-pyramidal and trigonal–bipyramidal geometries, respectively (Addison *et al.*, 1984 ▸). Further, consistent with this description is the observation that the benzyl­amino-N atoms are almost plumb to their respective N_4_ basal planes, as seen in the values of the amino-N—Zn—N(PC) angles collated in Table 1 ▸.

**Figure 2:**
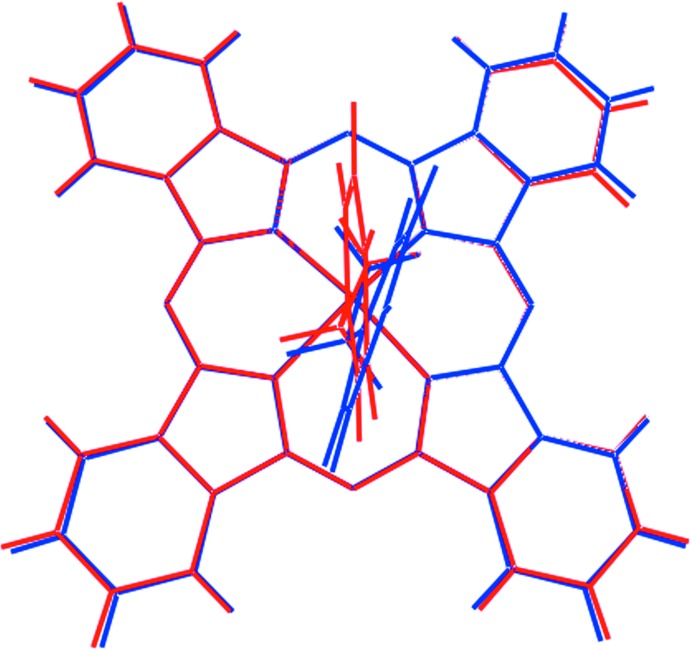
An overlay diagram of the two independent complex mol­ecules in (I). The Zn1- and Zn2-containing mol­ecules are shown as red and blue images, respectively. The mol­ecules have been overlapped so that the N_4_ square planes are coincident.

As seen from the overlay diagram in Fig. 2 ▸, the ZnPC cores are virtually identical in the independent mol­ecules. The obvious difference relates to the relative orientation of the benzyl­amine ligand with respect to the rest of the mol­ecule. While the Zn—N(amino)—C(methyl­ene)—C(phen­yl) torsion angles of −178.2 (3) and 176.3 (3)° are very similar for the two mol­ecules, the N(amino)—C(methyl­ene)—C(phen­yl)—C(phen­yl) torsion angles of −153.6 (4) and 26.7 (7) [Zn1-containing mol­ecule] and −178.8 (4) and 1.9 (7) [Zn2] differ.

A discussion of the uncoordinated benzyl­amine mol­ecules is found below in the *Database survey*.

## Supra­molecular features   

**Figure 3:**
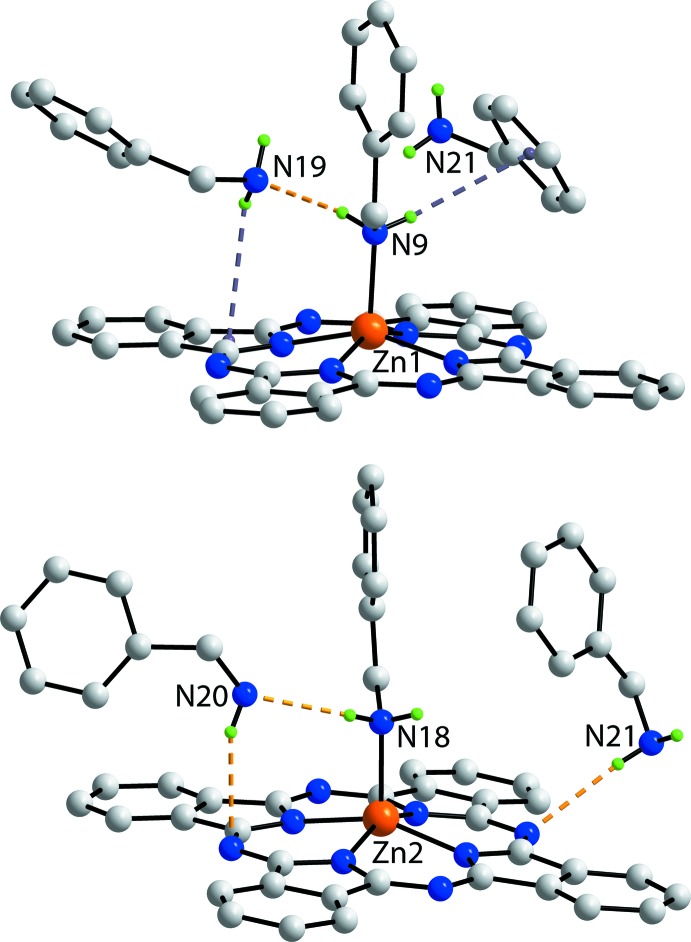
Detail of the N—H⋯N and N—H⋯π inter­actions, shown as orange and purple dashed lines, respectively, in the crystal packing of (I).

**Figure 4:**
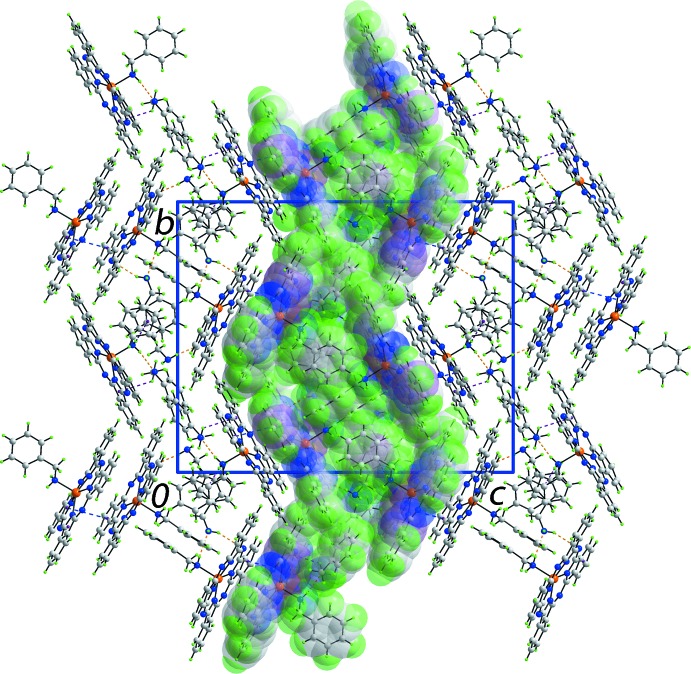
The unit-cell contents of (I), shown in projection down the *a* axis. Inter­molecular N—H⋯N, N—H⋯π and π–π inter­actions are shown as orange, purple and blue dashed lines, respectively. One supra­molecular layer sustained by N—H⋯N and N—H⋯π inter­actions has been highlighted in space-filling mode.

Based on the standard criteria incorporated within *PLATON* (Spek, 2009 ▸), the most notable directional inter­actions in the crystal packing of (I) are N—H⋯N hydrogen bonds, N—H⋯π inter­actions and face-to-face π–π inter­actions. These contacts involve six of the 10 available N—H atoms. The nature of the inter­actions involving amino-H atoms is highlighted in Fig. 3 ▸, and geometric parameters characterizing the inter­molecular inter­actions are given in Table 2 ▸. From the upper view of Fig. 3 ▸, it evident that the Zn1-bound benzyl­amine mol­ecule forms two N—H donor inter­actions, one being a conventional N—H⋯N hydrogen bond to the N19 atom of an uncoordinated benzyl­amine mol­ecule which is orientated to place one amine-H proximate to a five-membered NC_4_ ring, leading to a N—H⋯π(pyrrol­yl) inter­action. The second amine-H atom of the coordinating benzyl­amine mol­ecule forms an N—H⋯π(phen­yl) inter­action with a non-coordinating N21-benzyl­amine mol­ecule. For the Zn2-containing mol­ecule, the coordinating N18-benzyl­amine forms a donor N—H⋯N hydrogen bond to a non-coordinating N20-benzyl­amine mol­ecule which is folded to enable a donor N—H⋯N hydrogen bond to an exocyclic-N atom of the PC dianion. As seen from the lower view of Fig. 3 ▸, the second H atom of the N20-benzyl­amine mol­ecule does not form an inter­action within the standard distance criteria (Spek, 2009 ▸). The N21-benzyl­amine mol­ecule forms a donor N—H⋯N hydrogen bond to an exocyclic-N of the PC dianion. This mol­ecule functions as the bridge between the two complex mol­ecules and leads to the formation of supra­molecular layers in the *ab* plane. These have a zigzag topology and present the flat PC residues to the outside with the benzyl­amine mol­ecules, both coordinating and non-coordinating, in the inter-layer region. The layers stack along the *c* axis being connected by π–π inter­actions between pyrrolyl and fused-phenyl rings [inter-centroid (N6,C17, C18,C23,C24)⋯(C57–C62) distance = 3.593 (2) Å with an angle of inclination = 6.1 (2)°]. A view of the unit cell contents is shown in Fig. 4 ▸.

## Database survey   

A search of the Cambridge Structural Database (Groom & Allen, 2014 ▸) revealed five nondisordered literature precedents for ZnPC complexes being additionally coordinated by simple N-donors. In no examples were coordination numbers greater than five observed. There were two examples of simple 1:1 adducts, *i.e*. with 4-methyl­pridine (Kubiak *et al.*, 2007 ▸) and 1,8-di­aza­bicyclo­(4.5.0)undec-7-ene (Janczak *et al.*, 2011 ▸). In the 1:1 3-methyl­pyridin-2-amine adduct, there was an extra, non-coordinating 3-methyl­pyridin-2-amine mol­ecule in the structure (Janczak *et al.*, 2009 ▸). A similar situation pertains in the 1:1 adduct with 4-amino­pyridine but the non-coordinating solvent was tetra­hydro­furan in a ratio of 1:2 (Yang *et al.*, 2008 ▸). A particularly intriguing example was seen in the structure of ZnPC co-crystallized with pyrazine. The asymmetric unit comprises a binuclear mol­ecule arising from a μ_2_-pyrazine bridge, a mononuclear species where pyrazine is in the monodentate mode and non-coordinating pyrazine in a ratio 1:2:3 (Janczak & Kubiak, 2009 ▸). The basic structural motif for the aforementioned literature precedents matches that reported herein for (I). In terms of geometric parameters, as seen from Table 3 ▸, generally the Zn—N(PC) bond lengths span a narrow range, and are shorter that the Zn—N(donor) bond lengths with the notable exception being the adduct with 1,8-di­aza­bicyclo­(4.5.0)undec-7-ene (Janczak *et al.*, 2011 ▸). In this structure, the Zn—N(PC) bond lengths are systematically longer than in the other structures and the Zn—N(donor) bond shorter, consistent with a stronger coordinating ability of the 1,8-di­aza­bicyclo­(4.5.0)undec-7-ene ligand.

Two related Zn complexes are known with coordinated benzyl­amine (L), *i.e*. Zn(C_6_F_5_)_2_L_2_ (Mountford *et al.*, 2006 ▸) and in a tetra­hedral Zn complex featuring a tri-pyrazolyl ligand (Coquière *et al.*, 2008 ▸). In these, the Zn—N(benzyl­amine) bond lengths are 2.106 (4) and 2.106 (4) Å for the former, and 2.020 (4) Å in the latter, thereby being comparable and shorter, respectively, than the equivalent bonds in (I), Table 1 ▸.

**Figure 5:**
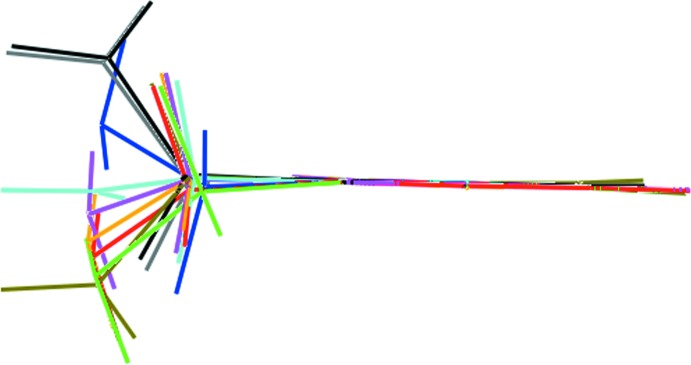
Overlay diagram of the non-coordinated benzyl­amine mol­ecules in (I) (red, green and blue images for the N19-, N20- and N21-containing mol­ecules, respectively), XAFTOP (orange), PUNMUH (cyan), EDOROE (pink) and EVUGIL (black, olive-green and grey for the three independent mol­ecules). The mol­ecules are overlapped so that the phenyl rings are coincident.

Finally, a few comments on the benzyl­amine mol­ecule which has now been characterized in its uncoordinated form in five crystal structures. From the *syn* and *anti*-C(phen­yl)—C(phen­yl)—C(benz­yl)—N(amine) torsion-angle data collated in Table 4 ▸ and the overlay diagram in Fig. 5 ▸, significant conformational flexibility is evident with respect to the relative orientation of the terminal amine group and the benzyl substituent. In a clathrate structure (Xiao *et al.*, 2010 ▸), where the mol­ecule is encapsulated within another large mol­ecule, an almost linear arrangement is seen. However, in the remaining examples twists up to 60° in the torsion angles are observed.

## Synthesis and crystallization   

Zinc phthalocyanine was prepared by a modification of a literature procedure (Bayo *et al.*, 2007 ▸). Phthalo­nitrile (4.30 g, 33.6 mmol) and zinc(II) acetate (2.50 g, 11.4 mmol) were refluxed in nitro­benzene (50.0 ml) for 4 h. The dark-violet crude product was filtered and washed with ethanol (30.0 ml) and acetone (15.0 ml). The product was purified by washing in a Soxhlet extractor with toluene (100 mL, 6 h), ethanol (200 mL, 9 h) and acetone (150 ml, 5 h). The purified dark-violet ZnPC solid was filtered and washed with 10% HCl (50 mL), 10% NaOH (50 mL) and water (10 ml). The product was dried in air (2.91 g, 60.0%). M.p.: > 503 K, IR (KBr, cm^−1^) ν 1608 *s* (C=N), 1584 *w* (C=C), 1377 *m*, 1334 *m*, 1285 *m*, 1164 *w*, 1118 *m*, 1088 *m*, 1060 *m*, 888 *w*, 878 *w*, 752 *m*, 728 *s*. Crystals of (I) were obtained from a solution in hot benzyl­amine and ethanol.

## Refinement   

Crystal data, data collection and structure refinement details are summarized in Table 5 ▸. Carbon-bound H atoms were placed in calculated positions (C—H = 0.95–0.99 Å) and were included in the refinement in the riding-model approximation, with *U*_iso_(H) set to 1.2*U*_eq_(C). The N-bound H atoms were treated similarly with N—H = 0.88 Å, and with *U*_iso_(H) = 1.2*U*_eq_(N). Each of three solvent benzyl­amine mol­ecules suffered from high thermal motion. In the final refinement the benzene rings were constrained to be regular hexa­gons (C—C = 1.39 Å) and their ADP’s restrained to be nearly isotropic using the ISOR command. Owing to poor agreement, two reflections, *i.e*. (0 3 2) and (4 0 14), were omitted from the final cycles of refinement.

## Supplementary Material

Crystal structure: contains datablock(s) I, global. DOI: 10.1107/S2056989015014280/hb7468sup1.cif

Structure factors: contains datablock(s) I. DOI: 10.1107/S2056989015014280/hb7468Isup2.hkl

CCDC reference: 1415614

Additional supporting information:  crystallographic information; 3D view; checkCIF report

## Figures and Tables

**Table 1:** Selected geometric parameters (, )

Zn1N2	2.033(3)	Zn2N11	2.034(3)
Zn1N4	2.045(3)	Zn2N13	2.031(3)
Zn1N6	2.025(3)	Zn2N15	2.032(3)
Zn1N8	2.037(3)	Zn2N17	2.031(3)
Zn1N9	2.105(3)	Zn2N18	2.117(3)
			
N6Zn1N2	153.99(13)	N17Zn2N13	154.73(13)
N6Zn1N8	87.96(12)	N17Zn2N15	87.06(12)
N2Zn1N8	86.50(12)	N13Zn2N15	87.56(12)
N6Zn1N4	86.35(12)	N17Zn2N11	87.61(12)
N2Zn1N4	87.12(12)	N13Zn2N11	87.19(12)
N8Zn1N4	152.94(13)	N15Zn2N11	155.64(13)
N2Zn1N9	104.47(13)	N11Zn2N18	102.55(13)
N4Zn1N9	105.55(13)	N13Zn2N18	106.46(13)
N6Zn1N9	101.55(13)	N15Zn2N18	101.75(13)
N8Zn1N9	101.51(13)	N17Zn2N18	98.81(13)

**Table 2:** Hydrogen-bond geometry (, ) *Cg*1 and *Cg*2 are the centroids of the C94C99 and N4/C9/C10/C15/C16 rings, respectively.

*D*H*A*	*D*H	H*A*	*D* *A*	*D*H*A*
N9H9*B*N19	0.88	2.22	3.093(5)	171
N18H18*A*N20	0.88	2.29	3.082(6)	150
N20H20*A*N16	0.88	2.52	3.277(6)	145
N21H21*B*N12	0.88	2.45	3.320(9)	169
N9H9*A* *Cg*1^i^	0.88	2.85	3.710(4)	165
N19H19*A* *Cg*2	0.88	2.91	3.407(4)	117

Symmetry code: (i) 
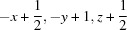
.

**Table 3:** Geometric data () for related Zn(PC)(N-donor) structures

N-donor	Range of ZnN(PC)	ZnN-donor	CSD refcode*^*a*^*	Reference
4-Methylpyridine	2.0061(16)2.0337(15)	2.1661(14)	WIJZEV	Kubiak *et al.* (2007 ▸)
1,8-Diazabicyclo[4.5.0]undec-7-ene	2.055(3)2.072(3)	2.064(3)	OPEVIP	Janczak *et al.* (2011 ▸)
3-Methylpyridin-2-amine	2.0325(18)2.037(2)	2.157(2)	MULYIC	Janczak *et al.* (2009 ▸)
4-Aminopyridine	2.0275(16)2.0343(17)	2.0916(16)	NOJGOJ	Yang *et al.* (2008 ▸)
Pyrazine*^*b*^*	2.009(3)2.012(3)	2.178(3)	KUHWIU	Janczak Kubiak (2009 ▸)
Pyrazine*^*c*^*	1.997(2)2.005(2)	2.207(2)		
Benzylamine	2.025(3)2.045(3)	2.105(3)2.117(3)		This work

Notes: (*a*) Groom Allen (2014 ▸); (*b*) mononuclear molecule; (*c*) centrosymmetric binuclear molecule.

**Table 4:** Summary of *syn* and *anti*-*C*(phenyl)C(phenyl)C(benzyl)N(amine) torsion angles () in structures having non-coordinating benzylamine molecules

Compound	*syn*-CCCN	*anti*-CCCN	CSD refcode*^*a*^*	Reference
Parent	31.5(3)	149.3(2)	XAFTOP	Nayak *et al.* (2010 ▸)
Clathrate	7.0(7)	174.0(5)	PUNMUH	Xiao *et al.* (2010 ▸)
Co-crystal	17.1(2)	163.81(14)	EDOROE	Suzuki Yatsugi (2002 ▸)
Co-crystal*^*b*^*	59.5(6)	118.4(5)	EVUGIL	Bourne *et al.* (2004 ▸)
	55.4(3)	125.8(3)		
	59.0(3)	122.0(3)		
Co-crystal*^*b*^*	28.2(5)	147.6(3)		This work
	35.1(14)	140.2(9)		
	28.7(10)	154.2(6)		

Notes: (*a*) Groom Allen (2014 ▸); (*b*) three independent benzylamine molecules.

**Table 5:** Experimental details

Crystal data	
Chemical formula	2[Zn(C_32_H_16_N_8_)(C_7_H_9_N)]3C_7_H_9_N
*M*_r_	1691.60
Crystal system, space group	Orthorhombic, *P*2_1_2_1_2_1_
Temperature (K)	100
*a*, *b*, *c* ()	12.3444(1), 22.7302(1), 28.2140(2)
*V* (^3^)	7916.59(9)
*Z*	4
Radiation type	Cu *K*
(mm^1^)	1.27
Crystal size (mm)	0.30 0.20 0.05

Data collection	
Diffractometer	Agilent SuperNova Dual with an Atlas detector
Absorption correction	Multi-scan (*CrysAlis PRO*; Agilent, 2013 ▸)
*T*_min_, *T*_max_	0.776, 1.000
No. of measured, independent and observed [*I* > 2(*I*)] reflections	45901, 16493, 16131
*R*_int_	0.027
(sin /)_max_ (^1^)	0.631

Refinement	
*R*[*F* ^2^ > 2(*F* ^2^)], *wR*(*F* ^2^), *S*	0.042, 0.114, 1.05
No. of reflections	16493
No. of parameters	1063
No. of restraints	144
H-atom treatment	H-atom parameters constrained
_max_, _min_ (e ^3^)	0.84, 0.50
Absolute structure	Flack *x* determined using 7057 quotients [(*I* ^+^)(*I* )]/[(*I* ^+^)+(*I* )] (Parsons *et al.*, 2013 ▸)
Absolute structure parameter	0.009(6)

Computer programs: *CrysAlis PRO* (Agilent, 2013 ▸), *SHELXS97* (Sheldrick, 2008 ▸), *SHELXL2014* (Sheldrick, 2015 ▸), *ORTEP-3 for Windows* (Farrugia, 2012 ▸), *QMol* (Gans Shalloway, 2001 ▸), *DIAMOND* (Brandenburg, 2006 ▸) and *publCIF* (Westrip, 2010 ▸).

## supplementary crystallographic information

### Crystal data

**Table:**

2[Zn(C_32_H_16_N_8_)(C_7_H_9_N)]·3C_7_H_9_N	*D*_x_ = 1.419 Mg m^−^^3^
*M**_r_* = 1691.60	Cu *K*α radiation, λ = 1.54184 Å
Orthorhombic, *P*2_1_2_1_2_1_	Cell parameters from 29184 reflections
*a* = 12.3444 (1) Å	θ = 3.1–76.4°
*b* = 22.7302 (1) Å	µ = 1.27 mm^−^^1^
*c* = 28.2140 (2) Å	*T* = 100 K
*V* = 7916.59 (9) Å^3^	Prism, blue
*Z* = 4	0.30 × 0.20 × 0.05 mm
*F*(000) = 3512	

### Data collection

**Table:**

Agilent SuperNova Dual with an Atlas detector diffractometer	16493 independent reflections
Radiation source: SuperNova (Cu) X-ray Source	16131 reflections with *I* > 2σ(*I*)
Mirror monochromator	*R*_int_ = 0.027
Detector resolution: 10.4041 pixels mm^-1^	θ_max_ = 76.6°, θ_min_ = 3.1°
ω scan	*h* = −9→15
Absorption correction: multi-scan (*CrysAlis PRO*; Agilent, 2013)	*k* = −28→25
*T*_min_ = 0.776, *T*_max_ = 1.000	*l* = −35→35
45901 measured reflections	

### Refinement

**Table:**

Refinement on *F*^2^	Hydrogen site location: inferred from neighbouring sites
Least-squares matrix: full	H-atom parameters constrained
*R*[*F*^2^ > 2σ(*F*^2^)] = 0.042	*w* = 1/[σ^2^(*F*_o_^2^) + (0.0628*P*)^2^ + 7.6343*P*] where *P* = (*F*_o_^2^ + 2*F*_c_^2^)/3
*wR*(*F*^2^) = 0.114	(Δ/σ)_max_ = 0.001
*S* = 1.05	Δρ_max_ = 0.84 e Å^−^^3^
16493 reflections	Δρ_min_ = −0.50 e Å^−^^3^
1063 parameters	Absolute structure: Flack *x* determined using 7057 quotients [(*I*^+^)-(*I*^-^)]/[(*I*^+^)+(*I*^-^)] (Parsons *et al.*, 2013)
144 restraints	Absolute structure parameter: −0.009 (6)

### Special details

**Table:**

Geometry. All e.s.d.'s (except the e.s.d. in the dihedral angle between two l.s. planes) are estimated using the full covariance matrix. The cell e.s.d.'s are taken into account individually in the estimation of e.s.d.'s in distances, angles and torsion angles; correlations between e.s.d.'s in cell parameters are only used when they are defined by crystal symmetry. An approximate (isotropic) treatment of cell e.s.d.'s is used for estimating e.s.d.'s involving l.s. planes.

### Fractional atomic coordinates and isotropic or equivalent isotropic displacement parameters (Å^2^)

**Table:**

	*x*	*y*	*z*	*U*_iso_*/*U*_eq_	
Zn1	0.20862 (4)	0.42414 (2)	0.80233 (2)	0.01424 (11)	
Zn2	0.40263 (4)	0.39302 (2)	0.62074 (2)	0.01546 (11)	
N1	0.2812 (2)	0.53377 (13)	0.72557 (10)	0.0149 (6)	
N2	0.3399 (2)	0.44202 (13)	0.76127 (11)	0.0152 (6)	
N3	0.4559 (2)	0.36031 (13)	0.78398 (11)	0.0163 (6)	
N4	0.2737 (2)	0.34238 (13)	0.81266 (11)	0.0161 (6)	
N5	0.1209 (3)	0.29345 (13)	0.84997 (11)	0.0172 (6)	
N6	0.0633 (2)	0.38642 (13)	0.81593 (11)	0.0162 (6)	
N7	−0.0552 (3)	0.46415 (14)	0.78732 (11)	0.0180 (6)	
N8	0.1287 (2)	0.48558 (13)	0.76310 (11)	0.0156 (6)	
N9	0.2373 (3)	0.47052 (14)	0.86568 (11)	0.0214 (6)	
H9A	0.1752	0.4847	0.8759	0.026*	
H9B	0.2608	0.4455	0.8871	0.026*	
N10	0.6660 (2)	0.35000 (14)	0.63252 (11)	0.0176 (6)	
N11	0.5495 (2)	0.42999 (14)	0.60719 (11)	0.0166 (6)	
N12	0.4927 (3)	0.52472 (13)	0.57673 (11)	0.0188 (6)	
N13	0.3375 (2)	0.47406 (13)	0.61032 (11)	0.0170 (6)	
N14	0.1545 (2)	0.45564 (13)	0.63831 (11)	0.0160 (6)	
N15	0.2700 (2)	0.37365 (13)	0.66045 (11)	0.0168 (6)	
N16	0.3292 (3)	0.28214 (13)	0.69605 (12)	0.0192 (6)	
N17	0.4821 (2)	0.33021 (14)	0.65831 (11)	0.0177 (6)	
N18	0.3722 (3)	0.34432 (15)	0.55818 (12)	0.0250 (7)	
H18A	0.3346	0.3127	0.5654	0.030*	
H18B	0.3330	0.3658	0.5387	0.030*	
N19	0.3192 (3)	0.37141 (17)	0.93171 (13)	0.0307 (8)	
H19A	0.2929	0.3367	0.9241	0.037*	
H19B	0.3000	0.3803	0.9609	0.037*	
N20	0.3271 (6)	0.2178 (2)	0.5921 (2)	0.0707 (17)	
H20A	0.3042	0.2232	0.6213	0.085*	
H20B	0.3984	0.2176	0.5917	0.085*	
N21	0.5421 (7)	0.5838 (4)	0.4713 (3)	0.107 (3)	
H21A	0.4848	0.5961	0.4556	0.128*	
H21B	0.5212	0.5656	0.4972	0.128*	
C1	0.3548 (3)	0.49267 (15)	0.73636 (12)	0.0141 (6)	
C2	0.4687 (3)	0.49656 (16)	0.72149 (13)	0.0158 (7)	
C3	0.5271 (3)	0.53928 (15)	0.69741 (14)	0.0180 (7)	
H3	0.4928	0.5738	0.6857	0.022*	
C4	0.6378 (3)	0.52986 (17)	0.69096 (14)	0.0202 (7)	
H4	0.6802	0.5590	0.6755	0.024*	
C5	0.6876 (3)	0.47796 (17)	0.70706 (13)	0.0202 (7)	
H5	0.7628	0.4722	0.7018	0.024*	
C6	0.6283 (3)	0.43491 (16)	0.73063 (13)	0.0189 (7)	
H6	0.6615	0.3996	0.7412	0.023*	
C7	0.5188 (3)	0.44533 (15)	0.73814 (13)	0.0159 (7)	
C8	0.4358 (3)	0.41171 (15)	0.76301 (12)	0.0152 (7)	
C9	0.3805 (3)	0.32860 (15)	0.80602 (13)	0.0162 (6)	
C10	0.4031 (3)	0.27186 (15)	0.82796 (12)	0.0162 (6)	
C11	0.4980 (3)	0.23900 (16)	0.83262 (14)	0.0193 (7)	
H11	0.5641	0.2520	0.8189	0.023*	
C12	0.4928 (3)	0.18654 (16)	0.85802 (14)	0.0204 (7)	
H12	0.5566	0.1638	0.8624	0.025*	
C13	0.3946 (3)	0.16677 (15)	0.87730 (14)	0.0214 (7)	
H13	0.3933	0.1307	0.8943	0.026*	
C14	0.2994 (3)	0.19848 (16)	0.87211 (13)	0.0186 (7)	
H14	0.2329	0.1845	0.8847	0.022*	
C15	0.3053 (3)	0.25219 (15)	0.84744 (13)	0.0172 (7)	
C16	0.2247 (3)	0.29752 (15)	0.83693 (12)	0.0164 (7)	
C17	0.0471 (3)	0.33448 (16)	0.83940 (13)	0.0158 (6)	
C18	−0.0681 (3)	0.32806 (16)	0.84993 (13)	0.0172 (7)	
C19	−0.1279 (3)	0.28465 (16)	0.87336 (14)	0.0196 (7)	
H19	−0.0935	0.2511	0.8867	0.023*	
C20	−0.2389 (3)	0.29217 (17)	0.87647 (15)	0.0237 (8)	
H20	−0.2814	0.2634	0.8923	0.028*	
C21	−0.2901 (3)	0.34192 (18)	0.85654 (14)	0.0239 (8)	
H21	−0.3666	0.3458	0.8589	0.029*	
C22	−0.2311 (3)	0.38496 (17)	0.83374 (14)	0.0201 (7)	
H22	−0.2657	0.4185	0.8206	0.024*	
C23	−0.1191 (3)	0.37770 (15)	0.83056 (13)	0.0169 (7)	
C24	−0.0345 (3)	0.41340 (16)	0.80939 (13)	0.0177 (7)	
C25	0.0208 (3)	0.49650 (16)	0.76623 (13)	0.0169 (7)	
C26	−0.0035 (3)	0.55148 (15)	0.74165 (13)	0.0166 (7)	
C27	−0.1008 (3)	0.58189 (16)	0.73544 (13)	0.0187 (7)	
H27	−0.1672	0.5674	0.7478	0.022*	
C28	−0.0958 (3)	0.63464 (16)	0.71021 (13)	0.0207 (7)	
H28	−0.1604	0.6564	0.7049	0.025*	
C29	0.0024 (3)	0.65614 (16)	0.69253 (13)	0.0203 (7)	
H29	0.0031	0.6923	0.6756	0.024*	
C30	0.0988 (3)	0.62576 (15)	0.69916 (13)	0.0170 (6)	
H30	0.1653	0.6406	0.6871	0.020*	
C31	0.0948 (3)	0.57301 (15)	0.72392 (12)	0.0158 (6)	
C32	0.1772 (3)	0.52946 (15)	0.73773 (12)	0.0144 (6)	
C33	0.3149 (4)	0.5191 (2)	0.86246 (16)	0.0353 (10)	
H33A	0.2872	0.5477	0.8390	0.042*	
H33B	0.3839	0.5032	0.8499	0.042*	
C34	0.3395 (3)	0.55204 (19)	0.90801 (15)	0.0266 (8)	
C35	0.3733 (4)	0.6101 (2)	0.90566 (16)	0.0288 (8)	
H35	0.3804	0.6288	0.8757	0.035*	
C36	0.3968 (4)	0.6412 (2)	0.94679 (18)	0.0357 (10)	
H36	0.4204	0.6809	0.9448	0.043*	
C37	0.3862 (4)	0.6146 (2)	0.99079 (16)	0.0343 (10)	
H37	0.4023	0.6361	1.0188	0.041*	
C38	0.3520 (4)	0.5568 (2)	0.99388 (16)	0.0321 (10)	
H38	0.3439	0.5386	1.0240	0.038*	
C39	0.3296 (4)	0.5255 (2)	0.95286 (15)	0.0298 (9)	
H39	0.3071	0.4856	0.9551	0.036*	
C40	0.6464 (3)	0.40162 (16)	0.61196 (12)	0.0171 (7)	
C41	0.7317 (3)	0.43827 (16)	0.59143 (12)	0.0178 (7)	
C42	0.8430 (3)	0.43036 (18)	0.58768 (13)	0.0216 (7)	
H42	0.8768	0.3950	0.5978	0.026*	
C43	0.9028 (3)	0.47652 (18)	0.56840 (14)	0.0245 (8)	
H43	0.9792	0.4727	0.5657	0.029*	
C44	0.8530 (3)	0.52840 (19)	0.55293 (15)	0.0251 (8)	
H44	0.8962	0.5590	0.5399	0.030*	
C45	0.7412 (3)	0.53600 (17)	0.55635 (14)	0.0213 (7)	
H45	0.7073	0.5712	0.5458	0.026*	
C46	0.6810 (3)	0.49007 (17)	0.57587 (13)	0.0188 (7)	
C47	0.5665 (3)	0.48336 (16)	0.58626 (13)	0.0176 (7)	
C48	0.3883 (3)	0.52005 (15)	0.58851 (13)	0.0171 (7)	
C49	0.3092 (3)	0.56648 (15)	0.57896 (12)	0.0166 (7)	
C50	0.3171 (3)	0.62178 (15)	0.55771 (13)	0.0185 (7)	
H50	0.3844	0.6363	0.5463	0.022*	
C51	0.2226 (3)	0.65480 (16)	0.55392 (13)	0.0202 (7)	
H51	0.2256	0.6926	0.5395	0.024*	
C52	0.1238 (3)	0.63388 (16)	0.57067 (13)	0.0195 (7)	
H52	0.0606	0.6572	0.5668	0.023*	
C53	0.1157 (3)	0.57936 (16)	0.59303 (12)	0.0180 (7)	
H53	0.0486	0.5656	0.6052	0.022*	
C54	0.2097 (3)	0.54599 (15)	0.59678 (12)	0.0157 (6)	
C55	0.2313 (3)	0.48778 (15)	0.61685 (12)	0.0153 (6)	
C56	0.1744 (3)	0.40352 (15)	0.65873 (13)	0.0164 (7)	
C57	0.0914 (3)	0.37041 (15)	0.68342 (12)	0.0170 (7)	
C58	−0.0178 (3)	0.38087 (16)	0.69201 (13)	0.0181 (7)	
H58	−0.0514	0.4161	0.6815	0.022*	
C59	−0.0764 (3)	0.33823 (18)	0.71641 (13)	0.0214 (7)	
H59	−0.1513	0.3443	0.7225	0.026*	
C60	−0.0262 (3)	0.28636 (17)	0.73210 (14)	0.0219 (7)	
H60	−0.0681	0.2576	0.7483	0.026*	
C61	0.0834 (3)	0.27611 (17)	0.72452 (13)	0.0206 (7)	
H61	0.1172	0.2412	0.7356	0.025*	
C62	0.1417 (3)	0.31873 (16)	0.70013 (14)	0.0187 (7)	
C63	0.2548 (3)	0.32293 (16)	0.68553 (13)	0.0180 (7)	
C64	0.4335 (3)	0.28689 (16)	0.68440 (13)	0.0175 (7)	
C65	0.5156 (3)	0.24327 (15)	0.69792 (14)	0.0180 (7)	
C66	0.5097 (3)	0.19127 (17)	0.72401 (14)	0.0214 (7)	
H66	0.4429	0.1776	0.7366	0.026*	
C67	0.6058 (4)	0.16010 (17)	0.73092 (14)	0.0251 (8)	
H67	0.6047	0.1246	0.7487	0.030*	
C68	0.7035 (3)	0.18029 (17)	0.71207 (14)	0.0238 (8)	
H68	0.7676	0.1580	0.7170	0.029*	
C69	0.7094 (3)	0.23215 (17)	0.68628 (14)	0.0216 (7)	
H69	0.7763	0.2459	0.6739	0.026*	
C70	0.6140 (3)	0.26331 (16)	0.67921 (13)	0.0186 (7)	
C71	0.5901 (3)	0.31830 (15)	0.65449 (13)	0.0170 (7)	
C72	0.4720 (4)	0.3266 (2)	0.53419 (16)	0.0315 (9)	
H72A	0.5179	0.3053	0.5573	0.038*	
H72B	0.5116	0.3627	0.5248	0.038*	
C73	0.4592 (4)	0.2880 (2)	0.49033 (15)	0.0285 (9)	
C74	0.5524 (4)	0.2696 (2)	0.46750 (16)	0.0329 (10)	
H74	0.6212	0.2789	0.4806	0.039*	
C75	0.5461 (5)	0.2380 (2)	0.42595 (17)	0.0378 (11)	
H75	0.6108	0.2266	0.4102	0.045*	
C76	0.4468 (5)	0.2228 (2)	0.40686 (17)	0.0413 (12)	
H76	0.4428	0.2019	0.3777	0.050*	
C77	0.3539 (5)	0.2383 (3)	0.43065 (18)	0.0444 (12)	
H77	0.2853	0.2266	0.4186	0.053*	
C78	0.3597 (4)	0.2711 (2)	0.47240 (18)	0.0400 (11)	
H78	0.2951	0.2819	0.4885	0.048*	
C79	0.4376 (4)	0.3700 (2)	0.92841 (17)	0.0329 (10)	
H79A	0.4649	0.4100	0.9358	0.040*	
H79B	0.4571	0.3616	0.8951	0.040*	
C80	0.4982 (2)	0.32636 (11)	0.95981 (9)	0.0303 (9)	
C81	0.6005 (2)	0.34133 (11)	0.97652 (11)	0.0357 (10)	
H81	0.6335	0.3771	0.9667	0.043*	
C82	0.6545 (2)	0.30400 (15)	1.00758 (11)	0.0462 (13)	
H82	0.7244	0.3142	1.0190	0.055*	
C83	0.6062 (3)	0.25171 (14)	1.02193 (11)	0.0468 (13)	
H83	0.6431	0.2262	1.0432	0.056*	
C84	0.5040 (3)	0.23673 (11)	1.00522 (11)	0.0435 (12)	
H84	0.4710	0.2010	1.0150	0.052*	
C85	0.4500 (2)	0.27406 (12)	0.97416 (11)	0.0326 (10)	
H85	0.3801	0.2638	0.9627	0.039*	
C86	0.2883 (10)	0.1639 (5)	0.5750 (4)	0.105 (3)	
H86A	0.3128	0.1595	0.5417	0.126*	
H86B	0.2082	0.1657	0.5745	0.126*	
C87	0.3219 (4)	0.10917 (16)	0.60217 (18)	0.078 (2)	
C88	0.3443 (4)	0.0648 (2)	0.56968 (13)	0.075 (2)	
H88	0.3327	0.0714	0.5368	0.090*	
C89	0.3835 (4)	0.01088 (18)	0.58529 (13)	0.0614 (17)	
H89	0.3988	−0.0194	0.5631	0.074*	
C90	0.4004 (3)	0.00127 (14)	0.63338 (15)	0.0484 (13)	
H90	0.4272	−0.0356	0.6440	0.058*	
C91	0.3780 (4)	0.04561 (19)	0.66587 (12)	0.0537 (14)	
H91	0.3895	0.0390	0.6987	0.064*	
C92	0.3388 (4)	0.09956 (17)	0.65026 (16)	0.074 (2)	
H92	0.3235	0.1299	0.6725	0.089*	
C93	0.6023 (8)	0.5445 (4)	0.4425 (3)	0.081 (2)	
H93A	0.6675	0.5316	0.4602	0.097*	
H93B	0.6270	0.5656	0.4138	0.097*	
C94	0.5418 (4)	0.49340 (19)	0.42842 (18)	0.074 (2)	
C95	0.5789 (4)	0.4687 (2)	0.38638 (17)	0.074 (2)	
H95	0.6366	0.4867	0.3695	0.089*	
C96	0.5316 (4)	0.4175 (2)	0.36905 (15)	0.084 (2)	
H96	0.5569	0.4006	0.3403	0.101*	
C97	0.4472 (4)	0.39111 (19)	0.39375 (16)	0.073 (2)	
H97	0.4148	0.3562	0.3819	0.088*	
C98	0.4101 (3)	0.4158 (2)	0.43579 (15)	0.0568 (15)	
H98	0.3524	0.3978	0.4527	0.068*	
C99	0.4574 (4)	0.46698 (19)	0.45312 (14)	0.069 (2)	
H99	0.4321	0.4839	0.4819	0.083*	

### Atomic displacement parameters (Å^2^)

**Table:**

	*U*^11^	*U*^22^	*U*^33^	*U*^12^	*U*^13^	*U*^23^
Zn1	0.0136 (2)	0.0130 (2)	0.0162 (2)	0.00064 (16)	−0.00001 (16)	0.00188 (17)
Zn2	0.0145 (2)	0.0136 (2)	0.0184 (2)	0.00129 (17)	−0.00184 (18)	0.00110 (17)
N1	0.0151 (13)	0.0146 (13)	0.0151 (13)	−0.0009 (11)	−0.0005 (11)	−0.0015 (10)
N2	0.0133 (13)	0.0140 (13)	0.0182 (14)	0.0001 (11)	0.0002 (11)	0.0007 (11)
N3	0.0160 (14)	0.0148 (13)	0.0182 (14)	−0.0007 (11)	−0.0016 (11)	0.0018 (11)
N4	0.0148 (14)	0.0147 (13)	0.0187 (14)	0.0000 (11)	0.0006 (11)	0.0037 (11)
N5	0.0189 (15)	0.0164 (14)	0.0164 (14)	−0.0013 (11)	0.0003 (11)	0.0014 (11)
N6	0.0141 (13)	0.0143 (14)	0.0201 (14)	−0.0005 (11)	0.0016 (11)	0.0020 (11)
N7	0.0156 (14)	0.0159 (14)	0.0225 (15)	0.0009 (11)	0.0027 (12)	0.0023 (12)
N8	0.0140 (13)	0.0147 (13)	0.0181 (14)	0.0001 (11)	0.0002 (11)	0.0022 (11)
N9	0.0260 (16)	0.0219 (15)	0.0164 (14)	0.0009 (13)	−0.0001 (12)	−0.0022 (12)
N10	0.0168 (14)	0.0173 (14)	0.0187 (14)	0.0015 (11)	−0.0026 (11)	0.0002 (11)
N11	0.0155 (13)	0.0149 (14)	0.0194 (14)	0.0031 (11)	−0.0005 (11)	0.0015 (11)
N12	0.0187 (15)	0.0159 (14)	0.0218 (15)	0.0005 (12)	−0.0002 (12)	0.0022 (12)
N13	0.0159 (14)	0.0161 (14)	0.0190 (15)	0.0004 (11)	−0.0012 (11)	0.0010 (11)
N14	0.0183 (14)	0.0146 (14)	0.0151 (14)	0.0022 (11)	−0.0008 (11)	−0.0002 (11)
N15	0.0159 (14)	0.0119 (13)	0.0225 (15)	0.0001 (11)	−0.0019 (11)	0.0046 (11)
N16	0.0199 (14)	0.0162 (14)	0.0215 (15)	0.0012 (11)	−0.0036 (12)	0.0033 (12)
N17	0.0145 (14)	0.0164 (14)	0.0222 (15)	0.0019 (11)	−0.0021 (12)	0.0021 (12)
N18	0.0236 (16)	0.0263 (17)	0.0251 (17)	0.0052 (13)	−0.0043 (13)	−0.0085 (13)
N19	0.036 (2)	0.0345 (19)	0.0219 (16)	0.0031 (16)	−0.0021 (15)	0.0027 (14)
N20	0.084 (4)	0.052 (3)	0.076 (4)	−0.004 (3)	−0.020 (3)	−0.017 (3)
N21	0.104 (6)	0.100 (6)	0.117 (6)	0.023 (5)	0.001 (5)	0.041 (5)
C1	0.0137 (15)	0.0156 (15)	0.0131 (15)	−0.0019 (12)	0.0007 (12)	0.0007 (12)
C2	0.0152 (16)	0.0177 (16)	0.0144 (16)	−0.0005 (13)	0.0008 (13)	−0.0025 (13)
C3	0.0194 (17)	0.0148 (15)	0.0198 (17)	−0.0007 (13)	0.0004 (14)	0.0006 (13)
C4	0.0162 (16)	0.0218 (17)	0.0227 (18)	−0.0040 (14)	0.0022 (14)	−0.0005 (14)
C5	0.0135 (16)	0.0242 (18)	0.0230 (18)	−0.0015 (14)	0.0035 (13)	−0.0024 (14)
C6	0.0158 (16)	0.0200 (17)	0.0208 (17)	0.0011 (13)	0.0017 (13)	−0.0007 (13)
C7	0.0159 (16)	0.0143 (15)	0.0175 (16)	−0.0012 (13)	0.0006 (13)	−0.0007 (13)
C8	0.0136 (15)	0.0160 (16)	0.0160 (16)	0.0019 (12)	−0.0006 (12)	−0.0011 (13)
C9	0.0156 (16)	0.0151 (15)	0.0180 (16)	0.0033 (12)	−0.0011 (13)	0.0008 (13)
C10	0.0178 (16)	0.0127 (15)	0.0181 (16)	0.0007 (13)	−0.0014 (14)	−0.0003 (12)
C11	0.0187 (17)	0.0148 (16)	0.0244 (18)	0.0012 (13)	−0.0016 (14)	−0.0012 (14)
C12	0.0224 (18)	0.0149 (16)	0.0240 (18)	0.0044 (14)	−0.0035 (15)	0.0003 (14)
C13	0.0277 (18)	0.0127 (15)	0.0239 (17)	0.0022 (14)	−0.0058 (16)	0.0019 (13)
C14	0.0218 (17)	0.0162 (16)	0.0177 (16)	−0.0015 (14)	−0.0011 (14)	0.0009 (13)
C15	0.0194 (17)	0.0133 (15)	0.0188 (16)	0.0022 (13)	−0.0014 (14)	−0.0006 (13)
C16	0.0196 (17)	0.0126 (15)	0.0170 (16)	0.0001 (13)	−0.0010 (13)	0.0028 (12)
C17	0.0161 (16)	0.0153 (16)	0.0161 (16)	−0.0008 (13)	0.0015 (12)	0.0010 (13)
C18	0.0167 (16)	0.0168 (16)	0.0182 (16)	0.0012 (13)	0.0019 (13)	0.0021 (13)
C19	0.0216 (17)	0.0169 (16)	0.0202 (17)	0.0004 (14)	0.0024 (14)	0.0022 (13)
C20	0.0232 (19)	0.0220 (18)	0.0258 (19)	−0.0046 (14)	0.0044 (16)	0.0030 (15)
C21	0.0168 (16)	0.0269 (19)	0.0280 (19)	−0.0013 (15)	0.0043 (15)	0.0015 (16)
C22	0.0164 (17)	0.0195 (17)	0.0243 (18)	0.0037 (13)	0.0019 (13)	0.0009 (14)
C23	0.0186 (18)	0.0153 (16)	0.0170 (16)	0.0006 (13)	0.0022 (13)	0.0011 (12)
C24	0.0149 (15)	0.0168 (16)	0.0213 (17)	0.0003 (13)	0.0007 (13)	0.0013 (13)
C25	0.0161 (16)	0.0161 (16)	0.0186 (17)	−0.0011 (13)	0.0001 (13)	0.0004 (13)
C26	0.0191 (17)	0.0141 (15)	0.0164 (16)	0.0009 (13)	−0.0019 (13)	0.0000 (13)
C27	0.0157 (15)	0.0152 (15)	0.0251 (17)	0.0020 (14)	−0.0021 (14)	0.0013 (14)
C28	0.0202 (17)	0.0178 (16)	0.0242 (18)	0.0050 (14)	−0.0033 (15)	−0.0001 (14)
C29	0.0277 (19)	0.0145 (16)	0.0187 (17)	0.0021 (14)	−0.0011 (15)	0.0016 (13)
C30	0.0187 (15)	0.0146 (15)	0.0177 (15)	−0.0016 (13)	−0.0012 (14)	0.0009 (13)
C31	0.0181 (15)	0.0146 (15)	0.0149 (15)	0.0012 (14)	−0.0030 (13)	−0.0016 (13)
C32	0.0161 (16)	0.0126 (15)	0.0146 (15)	−0.0005 (13)	−0.0030 (12)	−0.0009 (12)
C33	0.046 (3)	0.036 (2)	0.024 (2)	−0.017 (2)	0.0026 (18)	−0.0026 (18)
C34	0.029 (2)	0.029 (2)	0.0219 (19)	−0.0034 (16)	−0.0025 (16)	−0.0015 (16)
C35	0.030 (2)	0.029 (2)	0.028 (2)	−0.0023 (17)	−0.0021 (16)	−0.0029 (17)
C36	0.039 (2)	0.028 (2)	0.040 (2)	−0.0055 (19)	−0.004 (2)	−0.0070 (18)
C37	0.036 (2)	0.041 (2)	0.026 (2)	−0.004 (2)	−0.0049 (18)	−0.0098 (18)
C38	0.035 (2)	0.040 (2)	0.021 (2)	−0.0019 (19)	−0.0089 (17)	−0.0013 (17)
C39	0.037 (2)	0.029 (2)	0.023 (2)	−0.0036 (18)	−0.0028 (17)	0.0009 (16)
C40	0.0163 (16)	0.0192 (16)	0.0157 (16)	0.0012 (13)	−0.0018 (13)	−0.0010 (13)
C41	0.0183 (17)	0.0207 (17)	0.0144 (15)	−0.0013 (13)	−0.0003 (13)	−0.0005 (13)
C42	0.0182 (17)	0.0255 (19)	0.0212 (17)	0.0023 (14)	−0.0022 (14)	−0.0004 (15)
C43	0.0162 (16)	0.030 (2)	0.0275 (19)	−0.0010 (16)	0.0016 (15)	0.0014 (16)
C44	0.0200 (18)	0.030 (2)	0.0255 (19)	−0.0049 (16)	0.0017 (15)	0.0039 (16)
C45	0.0212 (18)	0.0225 (18)	0.0201 (17)	0.0003 (14)	0.0007 (14)	0.0018 (14)
C46	0.0173 (17)	0.0231 (18)	0.0161 (16)	0.0018 (14)	0.0002 (13)	0.0012 (14)
C47	0.0163 (16)	0.0197 (17)	0.0167 (16)	0.0004 (13)	0.0013 (13)	−0.0005 (13)
C48	0.0197 (17)	0.0122 (15)	0.0195 (16)	0.0001 (13)	−0.0025 (13)	0.0001 (12)
C49	0.0188 (16)	0.0162 (16)	0.0149 (15)	−0.0004 (13)	−0.0019 (13)	0.0009 (12)
C50	0.0201 (17)	0.0160 (16)	0.0194 (16)	0.0004 (13)	−0.0014 (13)	0.0012 (13)
C51	0.0285 (19)	0.0147 (16)	0.0173 (16)	0.0031 (14)	−0.0033 (14)	0.0016 (13)
C52	0.0223 (18)	0.0160 (16)	0.0202 (17)	0.0067 (14)	−0.0033 (14)	−0.0003 (13)
C53	0.0191 (16)	0.0169 (16)	0.0180 (16)	0.0013 (14)	−0.0037 (13)	−0.0012 (13)
C54	0.0208 (16)	0.0134 (15)	0.0130 (15)	−0.0008 (13)	−0.0014 (13)	0.0009 (12)
C55	0.0156 (16)	0.0142 (15)	0.0162 (15)	0.0012 (12)	−0.0018 (13)	0.0005 (13)
C56	0.0160 (15)	0.0160 (16)	0.0170 (16)	0.0014 (13)	−0.0019 (13)	−0.0003 (12)
C57	0.0197 (17)	0.0156 (15)	0.0157 (15)	−0.0015 (14)	−0.0020 (13)	−0.0009 (12)
C58	0.0190 (16)	0.0187 (16)	0.0167 (16)	0.0016 (13)	−0.0004 (13)	−0.0003 (13)
C59	0.0190 (17)	0.0262 (18)	0.0189 (17)	−0.0001 (14)	−0.0007 (13)	−0.0006 (14)
C60	0.0224 (18)	0.0236 (18)	0.0196 (18)	−0.0044 (15)	0.0013 (14)	0.0024 (14)
C61	0.0215 (18)	0.0198 (17)	0.0205 (17)	−0.0015 (14)	−0.0007 (15)	0.0015 (14)
C62	0.0173 (16)	0.0197 (17)	0.0190 (17)	0.0010 (13)	−0.0019 (14)	−0.0004 (14)
C63	0.0179 (16)	0.0164 (16)	0.0198 (17)	−0.0022 (13)	−0.0013 (13)	0.0018 (13)
C64	0.0181 (17)	0.0162 (16)	0.0183 (16)	0.0011 (13)	−0.0037 (13)	0.0015 (13)
C65	0.0182 (16)	0.0143 (15)	0.0214 (17)	0.0010 (13)	−0.0032 (14)	0.0006 (14)
C66	0.0253 (19)	0.0182 (17)	0.0206 (18)	0.0025 (15)	−0.0022 (15)	0.0024 (14)
C67	0.030 (2)	0.0197 (17)	0.0258 (19)	0.0039 (16)	−0.0043 (16)	0.0032 (14)
C68	0.0231 (18)	0.0199 (17)	0.0284 (19)	0.0078 (15)	−0.0061 (15)	0.0009 (15)
C69	0.0195 (17)	0.0202 (17)	0.0252 (18)	0.0023 (15)	−0.0048 (15)	−0.0010 (14)
C70	0.0201 (18)	0.0160 (16)	0.0196 (17)	0.0018 (14)	−0.0027 (14)	−0.0014 (13)
C71	0.0157 (16)	0.0159 (16)	0.0193 (16)	0.0024 (13)	−0.0043 (13)	−0.0007 (13)
C72	0.026 (2)	0.040 (2)	0.029 (2)	0.0031 (18)	−0.0020 (16)	−0.0124 (18)
C73	0.033 (2)	0.031 (2)	0.0219 (19)	0.0009 (17)	−0.0016 (16)	−0.0057 (16)
C74	0.038 (2)	0.035 (2)	0.026 (2)	0.0081 (19)	0.0022 (18)	−0.0046 (18)
C75	0.055 (3)	0.033 (2)	0.026 (2)	0.011 (2)	0.006 (2)	−0.0030 (18)
C76	0.067 (4)	0.030 (2)	0.027 (2)	−0.001 (2)	0.000 (2)	−0.0061 (18)
C77	0.055 (3)	0.047 (3)	0.031 (2)	−0.009 (2)	−0.007 (2)	−0.011 (2)
C78	0.037 (2)	0.049 (3)	0.034 (2)	−0.005 (2)	−0.003 (2)	−0.016 (2)
C79	0.036 (2)	0.034 (2)	0.029 (2)	0.0029 (18)	−0.0010 (18)	0.0044 (17)
C80	0.035 (2)	0.034 (2)	0.0219 (18)	0.0098 (18)	0.0002 (16)	−0.0065 (16)
C81	0.039 (2)	0.041 (2)	0.027 (2)	0.008 (2)	−0.0002 (19)	−0.0074 (18)
C82	0.046 (3)	0.059 (3)	0.034 (2)	0.021 (2)	−0.006 (2)	−0.011 (2)
C83	0.055 (3)	0.054 (3)	0.032 (2)	0.032 (3)	−0.004 (2)	−0.004 (2)
C84	0.061 (3)	0.035 (2)	0.034 (2)	0.023 (2)	0.005 (2)	−0.0020 (19)
C85	0.043 (2)	0.030 (2)	0.0247 (19)	0.0122 (18)	−0.0005 (18)	−0.0063 (16)
C86	0.108 (6)	0.089 (5)	0.118 (6)	−0.003 (5)	−0.025 (5)	0.003 (5)
C87	0.073 (4)	0.058 (4)	0.104 (5)	−0.015 (3)	−0.043 (4)	0.013 (4)
C88	0.078 (4)	0.076 (4)	0.071 (4)	−0.027 (4)	−0.025 (3)	0.015 (3)
C89	0.054 (3)	0.065 (4)	0.065 (4)	−0.019 (3)	−0.006 (3)	−0.001 (3)
C90	0.039 (2)	0.040 (3)	0.065 (3)	−0.007 (2)	−0.001 (2)	0.012 (2)
C91	0.046 (3)	0.060 (3)	0.055 (3)	−0.006 (3)	−0.010 (2)	0.013 (3)
C92	0.066 (4)	0.062 (4)	0.094 (5)	0.006 (3)	−0.032 (4)	−0.024 (3)
C93	0.076 (4)	0.081 (4)	0.085 (4)	0.004 (4)	0.018 (4)	0.018 (4)
C94	0.072 (4)	0.081 (4)	0.069 (4)	−0.007 (4)	−0.018 (3)	0.019 (3)
C95	0.069 (4)	0.095 (5)	0.059 (4)	0.004 (4)	0.006 (3)	0.026 (4)
C96	0.079 (4)	0.104 (5)	0.071 (4)	0.001 (4)	0.005 (4)	0.026 (4)
C97	0.074 (4)	0.074 (4)	0.071 (4)	0.004 (3)	0.002 (3)	0.025 (3)
C98	0.061 (3)	0.060 (3)	0.049 (3)	0.015 (3)	−0.006 (3)	0.004 (3)
C99	0.057 (3)	0.073 (4)	0.077 (4)	−0.007 (3)	−0.012 (3)	0.043 (3)

### Geometric parameters (Å, º)

**Table:**

Zn1—N2	2.033 (3)	C36—C37	1.387 (7)
Zn1—N4	2.045 (3)	C36—H36	0.9500
Zn1—N6	2.025 (3)	C37—C38	1.383 (7)
Zn1—N8	2.037 (3)	C37—H37	0.9500
Zn1—N9	2.105 (3)	C38—C39	1.386 (6)
Zn2—N11	2.034 (3)	C38—H38	0.9500
Zn2—N13	2.031 (3)	C39—H39	0.9500
Zn2—N15	2.032 (3)	C40—C41	1.462 (5)
Zn2—N17	2.031 (3)	C41—C42	1.389 (5)
Zn2—N18	2.117 (3)	C41—C46	1.404 (5)
N1—C32	1.333 (5)	C42—C43	1.394 (6)
N1—C1	1.338 (5)	C42—H42	0.9500
N2—C1	1.361 (4)	C43—C44	1.400 (6)
N2—C8	1.371 (4)	C43—H43	0.9500
N3—C9	1.331 (5)	C44—C45	1.394 (5)
N3—C8	1.333 (5)	C44—H44	0.9500
N4—C9	1.367 (4)	C45—C46	1.395 (5)
N4—C16	1.369 (5)	C45—H45	0.9500
N5—C16	1.337 (5)	C46—C47	1.451 (5)
N5—C17	1.337 (5)	C48—C49	1.463 (5)
N6—C24	1.366 (5)	C49—C50	1.396 (5)
N6—C17	1.369 (5)	C49—C54	1.406 (5)
N7—C25	1.333 (5)	C50—C51	1.392 (5)
N7—C24	1.336 (5)	C50—H50	0.9500
N8—C25	1.358 (5)	C51—C52	1.392 (6)
N8—C32	1.366 (5)	C51—H51	0.9500
N9—C33	1.465 (5)	C52—C53	1.394 (5)
N9—H9A	0.8800	C52—H52	0.9500
N9—H9B	0.8800	C53—C54	1.391 (5)
N10—C40	1.331 (5)	C53—H53	0.9500
N10—C71	1.335 (5)	C54—C55	1.464 (5)
N11—C47	1.365 (5)	C56—C57	1.449 (5)
N11—C40	1.366 (5)	C57—C58	1.390 (5)
N12—C48	1.335 (5)	C57—C62	1.410 (5)
N12—C47	1.336 (5)	C58—C59	1.392 (5)
N13—C55	1.360 (4)	C58—H58	0.9500
N13—C48	1.366 (5)	C59—C60	1.404 (6)
N14—C56	1.340 (5)	C59—H59	0.9500
N14—C55	1.342 (5)	C60—C61	1.389 (6)
N15—C56	1.363 (5)	C60—H60	0.9500
N15—C63	1.366 (5)	C61—C62	1.390 (5)
N16—C64	1.334 (5)	C61—H61	0.9500
N16—C63	1.338 (5)	C62—C63	1.458 (5)
N17—C71	1.366 (5)	C64—C65	1.468 (5)
N17—C64	1.368 (5)	C65—C66	1.395 (5)
N18—C72	1.462 (5)	C65—C70	1.400 (5)
N18—H18A	0.8800	C66—C67	1.396 (6)
N18—H18B	0.8800	C66—H66	0.9500
N19—C79	1.465 (6)	C67—C68	1.396 (6)
N19—H19A	0.8800	C67—H67	0.9500
N19—H19B	0.8800	C68—C69	1.387 (5)
N20—C86	1.401 (11)	C68—H68	0.9500
N20—H20A	0.8800	C69—C70	1.389 (5)
N20—H20B	0.8800	C69—H69	0.9500
N21—C93	1.418 (12)	C70—C71	1.461 (5)
N21—H21A	0.8800	C72—C73	1.526 (6)
N21—H21B	0.8800	C72—H72A	0.9900
C1—C2	1.470 (5)	C72—H72B	0.9900
C2—C3	1.387 (5)	C73—C78	1.382 (7)
C2—C7	1.400 (5)	C73—C74	1.383 (6)
C3—C4	1.396 (5)	C74—C75	1.377 (6)
C3—H3	0.9500	C74—H74	0.9500
C4—C5	1.406 (5)	C75—C76	1.384 (8)
C4—H4	0.9500	C75—H75	0.9500
C5—C6	1.391 (5)	C76—C77	1.375 (8)
C5—H5	0.9500	C76—H76	0.9500
C6—C7	1.388 (5)	C77—C78	1.397 (7)
C6—H6	0.9500	C77—H77	0.9500
C7—C8	1.459 (5)	C78—H78	0.9500
C9—C10	1.458 (5)	C79—C80	1.527 (5)
C10—C11	1.395 (5)	C79—H79A	0.9900
C10—C15	1.401 (5)	C79—H79B	0.9900
C11—C12	1.393 (5)	C80—C81	1.3900
C11—H11	0.9500	C80—C85	1.3900
C12—C13	1.403 (6)	C81—C82	1.3900
C12—H12	0.9500	C81—H81	0.9500
C13—C14	1.386 (5)	C82—C83	1.3900
C13—H13	0.9500	C82—H82	0.9500
C14—C15	1.407 (5)	C83—C84	1.3900
C14—H14	0.9500	C83—H83	0.9500
C15—C16	1.463 (5)	C84—C85	1.3900
C17—C18	1.460 (5)	C84—H84	0.9500
C18—C19	1.399 (5)	C85—H85	0.9500
C18—C23	1.403 (5)	C86—C87	1.520 (11)
C19—C20	1.384 (5)	C86—H86A	0.9900
C19—H19	0.9500	C86—H86B	0.9900
C20—C21	1.412 (6)	C87—C88	1.3900
C20—H20	0.9500	C87—C92	1.3900
C21—C22	1.379 (5)	C88—C89	1.3900
C21—H21	0.9500	C88—H88	0.9500
C22—C23	1.395 (5)	C89—C90	1.3900
C22—H22	0.9500	C89—H89	0.9500
C23—C24	1.452 (5)	C90—C91	1.3900
C25—C26	1.460 (5)	C90—H90	0.9500
C26—C27	1.397 (5)	C91—C92	1.3900
C26—C31	1.402 (5)	C91—H91	0.9500
C27—C28	1.396 (5)	C92—H92	0.9500
C27—H27	0.9500	C93—C94	1.436 (10)
C28—C29	1.398 (6)	C93—H93A	0.9900
C28—H28	0.9500	C93—H93B	0.9900
C29—C30	1.389 (5)	C94—C95	1.3900
C29—H29	0.9500	C94—C99	1.3900
C30—C31	1.388 (5)	C95—C96	1.3900
C30—H30	0.9500	C95—H95	0.9500
C31—C32	1.472 (5)	C96—C97	1.3900
C33—C34	1.518 (6)	C96—H96	0.9500
C33—H33A	0.9900	C97—C98	1.3900
C33—H33B	0.9900	C97—H97	0.9500
C34—C35	1.385 (6)	C98—C99	1.3900
C34—C39	1.407 (6)	C98—H98	0.9500
C35—C36	1.390 (6)	C99—H99	0.9500
C35—H35	0.9500		
			
N6—Zn1—N2	153.99 (13)	N10—C40—N11	128.2 (3)
N6—Zn1—N8	87.96 (12)	N10—C40—C41	123.0 (3)
N2—Zn1—N8	86.50 (12)	N11—C40—C41	108.8 (3)
N6—Zn1—N4	86.35 (12)	C42—C41—C46	121.7 (4)
N2—Zn1—N4	87.12 (12)	C42—C41—C40	131.9 (4)
N8—Zn1—N4	152.94 (13)	C46—C41—C40	106.3 (3)
N2—Zn1—N9	104.47 (13)	C41—C42—C43	117.1 (4)
N4—Zn1—N9	105.55 (13)	C41—C42—H42	121.4
N6—Zn1—N9	101.55 (13)	C43—C42—H42	121.4
N8—Zn1—N9	101.51 (13)	C42—C43—C44	121.6 (4)
N17—Zn2—N13	154.73 (13)	C42—C43—H43	119.2
N17—Zn2—N15	87.06 (12)	C44—C43—H43	119.2
N13—Zn2—N15	87.56 (12)	C45—C44—C43	121.2 (4)
N17—Zn2—N11	87.61 (12)	C45—C44—H44	119.4
N13—Zn2—N11	87.19 (12)	C43—C44—H44	119.4
N15—Zn2—N11	155.64 (13)	C44—C45—C46	117.5 (4)
N11—Zn2—N18	102.55 (13)	C44—C45—H45	121.2
N13—Zn2—N18	106.46 (13)	C46—C45—H45	121.2
N15—Zn2—N18	101.75 (13)	C45—C46—C41	120.9 (3)
N17—Zn2—N18	98.81 (13)	C45—C46—C47	132.6 (4)
C32—N1—C1	123.0 (3)	C41—C46—C47	106.5 (3)
C1—N2—C8	109.1 (3)	N12—C47—N11	127.4 (3)
C1—N2—Zn1	124.8 (2)	N12—C47—C46	123.3 (3)
C8—N2—Zn1	124.6 (2)	N11—C47—C46	109.3 (3)
C9—N3—C8	123.5 (3)	N12—C48—N13	128.0 (3)
C9—N4—C16	108.9 (3)	N12—C48—C49	122.8 (3)
C9—N4—Zn1	124.6 (2)	N13—C48—C49	109.2 (3)
C16—N4—Zn1	125.1 (2)	C50—C49—C54	120.9 (3)
C16—N5—C17	122.9 (3)	C50—C49—C48	133.0 (3)
C24—N6—C17	108.9 (3)	C54—C49—C48	106.1 (3)
C24—N6—Zn1	124.5 (2)	C51—C50—C49	117.4 (3)
C17—N6—Zn1	125.9 (2)	C51—C50—H50	121.3
C25—N7—C24	123.3 (3)	C49—C50—H50	121.3
C25—N8—C32	109.3 (3)	C50—C51—C52	121.6 (3)
C25—N8—Zn1	124.4 (2)	C50—C51—H51	119.2
C32—N8—Zn1	125.0 (2)	C52—C51—H51	119.2
C33—N9—Zn1	115.8 (3)	C51—C52—C53	121.3 (3)
C33—N9—H9A	108.3	C51—C52—H52	119.3
Zn1—N9—H9A	108.3	C53—C52—H52	119.3
C33—N9—H9B	108.3	C54—C53—C52	117.3 (3)
Zn1—N9—H9B	108.3	C54—C53—H53	121.3
H9A—N9—H9B	107.4	C52—C53—H53	121.3
C40—N10—C71	123.4 (3)	C53—C54—C49	121.4 (3)
C47—N11—C40	109.1 (3)	C53—C54—C55	132.4 (3)
C47—N11—Zn2	125.8 (2)	C49—C54—C55	106.2 (3)
C40—N11—Zn2	124.5 (2)	N14—C55—N13	128.2 (3)
C48—N12—C47	123.5 (3)	N14—C55—C54	122.5 (3)
C55—N13—C48	109.2 (3)	N13—C55—C54	109.3 (3)
C55—N13—Zn2	124.7 (2)	N14—C56—N15	127.9 (3)
C48—N13—Zn2	125.3 (2)	N14—C56—C57	122.4 (3)
C56—N14—C55	123.1 (3)	N15—C56—C57	109.7 (3)
C56—N15—C63	108.6 (3)	C58—C57—C62	120.7 (3)
C56—N15—Zn2	124.8 (2)	C58—C57—C56	132.9 (3)
C63—N15—Zn2	125.4 (2)	C62—C57—C56	106.4 (3)
C64—N16—C63	123.5 (3)	C57—C58—C59	118.1 (3)
C71—N17—C64	109.1 (3)	C57—C58—H58	120.9
C71—N17—Zn2	124.7 (3)	C59—C58—H58	120.9
C64—N17—Zn2	125.1 (2)	C58—C59—C60	120.7 (4)
C72—N18—Zn2	112.4 (3)	C58—C59—H59	119.6
C72—N18—H18A	109.1	C60—C59—H59	119.6
Zn2—N18—H18A	109.1	C61—C60—C59	121.5 (4)
C72—N18—H18B	109.1	C61—C60—H60	119.3
Zn2—N18—H18B	109.1	C59—C60—H60	119.3
H18A—N18—H18B	107.9	C60—C61—C62	117.6 (4)
C79—N19—H19A	109.5	C60—C61—H61	121.2
C79—N19—H19B	109.5	C62—C61—H61	121.2
H19A—N19—H19B	109.5	C61—C62—C57	121.2 (3)
C86—N20—H20A	109.5	C61—C62—C63	133.0 (3)
C86—N20—H20B	109.5	C57—C62—C63	105.8 (3)
H20A—N20—H20B	109.5	N16—C63—N15	127.2 (3)
C93—N21—H21A	109.5	N16—C63—C62	123.3 (3)
C93—N21—H21B	109.5	N15—C63—C62	109.5 (3)
H21A—N21—H21B	109.5	N16—C64—N17	127.9 (3)
N1—C1—N2	128.0 (3)	N16—C64—C65	123.2 (3)
N1—C1—C2	122.8 (3)	N17—C64—C65	108.9 (3)
N2—C1—C2	109.1 (3)	C66—C65—C70	121.3 (3)
C3—C2—C7	121.1 (3)	C66—C65—C64	132.4 (4)
C3—C2—C1	132.8 (3)	C70—C65—C64	106.3 (3)
C7—C2—C1	106.1 (3)	C65—C66—C67	117.4 (4)
C2—C3—C4	117.8 (3)	C65—C66—H66	121.3
C2—C3—H3	121.1	C67—C66—H66	121.3
C4—C3—H3	121.1	C66—C67—C68	121.0 (4)
C3—C4—C5	121.0 (3)	C66—C67—H67	119.5
C3—C4—H4	119.5	C68—C67—H67	119.5
C5—C4—H4	119.5	C69—C68—C67	121.6 (4)
C6—C5—C4	121.0 (3)	C69—C68—H68	119.2
C6—C5—H5	119.5	C67—C68—H68	119.2
C4—C5—H5	119.5	C68—C69—C70	117.7 (4)
C7—C6—C5	117.7 (3)	C68—C69—H69	121.2
C7—C6—H6	121.1	C70—C69—H69	121.2
C5—C6—H6	121.1	C69—C70—C65	121.0 (3)
C6—C7—C2	121.4 (3)	C69—C70—C71	132.5 (4)
C6—C7—C8	131.9 (3)	C65—C70—C71	106.5 (3)
C2—C7—C8	106.6 (3)	N10—C71—N17	128.0 (3)
N3—C8—N2	128.1 (3)	N10—C71—C70	122.8 (3)
N3—C8—C7	122.8 (3)	N17—C71—C70	109.2 (3)
N2—C8—C7	109.1 (3)	N18—C72—C73	116.5 (4)
N3—C9—N4	127.9 (3)	N18—C72—H72A	108.2
N3—C9—C10	122.9 (3)	C73—C72—H72A	108.2
N4—C9—C10	109.3 (3)	N18—C72—H72B	108.2
C11—C10—C15	121.1 (3)	C73—C72—H72B	108.2
C11—C10—C9	132.4 (3)	H72A—C72—H72B	107.3
C15—C10—C9	106.5 (3)	C78—C73—C74	119.0 (4)
C12—C11—C10	117.9 (4)	C78—C73—C72	123.3 (4)
C12—C11—H11	121.0	C74—C73—C72	117.7 (4)
C10—C11—H11	121.0	C75—C74—C73	120.5 (5)
C11—C12—C13	120.9 (3)	C75—C74—H74	119.8
C11—C12—H12	119.5	C73—C74—H74	119.8
C13—C12—H12	119.5	C74—C75—C76	120.8 (5)
C14—C13—C12	121.7 (3)	C74—C75—H75	119.6
C14—C13—H13	119.2	C76—C75—H75	119.6
C12—C13—H13	119.2	C77—C76—C75	119.0 (4)
C13—C14—C15	117.4 (3)	C77—C76—H76	120.5
C13—C14—H14	121.3	C75—C76—H76	120.5
C15—C14—H14	121.3	C76—C77—C78	120.4 (5)
C10—C15—C14	121.0 (3)	C76—C77—H77	119.8
C10—C15—C16	106.4 (3)	C78—C77—H77	119.8
C14—C15—C16	132.6 (3)	C73—C78—C77	120.1 (5)
N5—C16—N4	127.8 (3)	C73—C78—H78	119.9
N5—C16—C15	123.2 (3)	C77—C78—H78	119.9
N4—C16—C15	109.0 (3)	N19—C79—C80	117.8 (4)
N5—C17—N6	127.6 (3)	N19—C79—H79A	107.9
N5—C17—C18	123.3 (3)	C80—C79—H79A	107.9
N6—C17—C18	109.1 (3)	N19—C79—H79B	107.9
C19—C18—C23	120.9 (3)	C80—C79—H79B	107.9
C19—C18—C17	132.9 (3)	H79A—C79—H79B	107.2
C23—C18—C17	106.1 (3)	C81—C80—C85	120.0
C20—C19—C18	117.8 (4)	C81—C80—C79	118.9 (3)
C20—C19—H19	121.1	C85—C80—C79	121.0 (3)
C18—C19—H19	121.1	C82—C81—C80	120.0
C19—C20—C21	121.1 (4)	C82—C81—H81	120.0
C19—C20—H20	119.4	C80—C81—H81	120.0
C21—C20—H20	119.4	C81—C82—C83	120.0
C22—C21—C20	121.1 (4)	C81—C82—H82	120.0
C22—C21—H21	119.4	C83—C82—H82	120.0
C20—C21—H21	119.4	C82—C83—C84	120.0
C21—C22—C23	118.0 (4)	C82—C83—H83	120.0
C21—C22—H22	121.0	C84—C83—H83	120.0
C23—C22—H22	121.0	C85—C84—C83	120.0
C22—C23—C18	121.0 (3)	C85—C84—H84	120.0
C22—C23—C24	132.4 (3)	C83—C84—H84	120.0
C18—C23—C24	106.7 (3)	C84—C85—C80	120.0
N7—C24—N6	128.3 (3)	C84—C85—H85	120.0
N7—C24—C23	122.4 (3)	C80—C85—H85	120.0
N6—C24—C23	109.2 (3)	N20—C86—C87	116.7 (8)
N7—C25—N8	128.3 (3)	N20—C86—H86A	108.1
N7—C25—C26	122.6 (3)	C87—C86—H86A	108.1
N8—C25—C26	109.1 (3)	N20—C86—H86B	108.1
C27—C26—C31	121.8 (3)	C87—C86—H86B	108.1
C27—C26—C25	131.3 (3)	H86A—C86—H86B	107.3
C31—C26—C25	106.9 (3)	C88—C87—C92	120.0
C28—C27—C26	116.8 (3)	C88—C87—C86	108.4 (6)
C28—C27—H27	121.6	C92—C87—C86	131.5 (6)
C26—C27—H27	121.6	C89—C88—C87	120.0
C29—C28—C27	121.4 (3)	C89—C88—H88	120.0
C29—C28—H28	119.3	C87—C88—H88	120.0
C27—C28—H28	119.3	C88—C89—C90	120.0
C30—C29—C28	121.4 (3)	C88—C89—H89	120.0
C30—C29—H29	119.3	C90—C89—H89	120.0
C28—C29—H29	119.3	C91—C90—C89	120.0
C31—C30—C29	117.9 (3)	C91—C90—H90	120.0
C31—C30—H30	121.1	C89—C90—H90	120.0
C29—C30—H30	121.1	C90—C91—C92	120.0
C30—C31—C26	120.8 (3)	C90—C91—H91	120.0
C30—C31—C32	133.6 (3)	C92—C91—H91	120.0
C26—C31—C32	105.6 (3)	C91—C92—C87	120.0
N1—C32—N8	127.6 (3)	C91—C92—H92	120.0
N1—C32—C31	123.2 (3)	C87—C92—H92	120.0
N8—C32—C31	109.1 (3)	N21—C93—C94	113.3 (8)
N9—C33—C34	116.8 (4)	N21—C93—H93A	108.9
N9—C33—H33A	108.1	C94—C93—H93A	108.9
C34—C33—H33A	108.1	N21—C93—H93B	108.9
N9—C33—H33B	108.1	C94—C93—H93B	108.9
C34—C33—H33B	108.1	H93A—C93—H93B	107.7
H33A—C33—H33B	107.3	C95—C94—C99	120.0
C35—C34—C39	118.5 (4)	C95—C94—C93	113.1 (5)
C35—C34—C33	119.3 (4)	C99—C94—C93	126.8 (5)
C39—C34—C33	122.2 (4)	C94—C95—C96	120.0
C34—C35—C36	120.5 (4)	C94—C95—H95	120.0
C34—C35—H35	119.7	C96—C95—H95	120.0
C36—C35—H35	119.7	C97—C96—C95	120.0
C37—C36—C35	120.4 (4)	C97—C96—H96	120.0
C37—C36—H36	119.8	C95—C96—H96	120.0
C35—C36—H36	119.8	C96—C97—C98	120.0
C38—C37—C36	120.0 (4)	C96—C97—H97	120.0
C38—C37—H37	120.0	C98—C97—H97	120.0
C36—C37—H37	120.0	C97—C98—C99	120.0
C37—C38—C39	119.7 (4)	C97—C98—H98	120.0
C37—C38—H38	120.2	C99—C98—H98	120.0
C39—C38—H38	120.2	C98—C99—C94	120.0
C38—C39—C34	120.9 (4)	C98—C99—H99	120.0
C38—C39—H39	119.5	C94—C99—H99	120.0
C34—C39—H39	119.5		
			
C32—N1—C1—N2	−1.5 (6)	C44—C45—C46—C41	0.0 (6)
C32—N1—C1—C2	177.8 (3)	C44—C45—C46—C47	177.5 (4)
C8—N2—C1—N1	178.3 (3)	C42—C41—C46—C45	−0.6 (6)
Zn1—N2—C1—N1	−15.3 (5)	C40—C41—C46—C45	176.9 (3)
C8—N2—C1—C2	−1.2 (4)	C42—C41—C46—C47	−178.7 (3)
Zn1—N2—C1—C2	165.2 (2)	C40—C41—C46—C47	−1.2 (4)
N1—C1—C2—C3	3.5 (6)	C48—N12—C47—N11	2.7 (6)
N2—C1—C2—C3	−177.0 (4)	C48—N12—C47—C46	−176.3 (4)
N1—C1—C2—C7	−178.4 (3)	C40—N11—C47—N12	−178.0 (4)
N2—C1—C2—C7	1.0 (4)	Zn2—N11—C47—N12	10.0 (5)
C7—C2—C3—C4	−0.9 (5)	C40—N11—C47—C46	1.1 (4)
C1—C2—C3—C4	176.9 (4)	Zn2—N11—C47—C46	−170.9 (2)
C2—C3—C4—C5	1.9 (6)	C45—C46—C47—N12	1.4 (7)
C3—C4—C5—C6	−1.1 (6)	C41—C46—C47—N12	179.2 (3)
C4—C5—C6—C7	−0.7 (5)	C45—C46—C47—N11	−177.7 (4)
C5—C6—C7—C2	1.8 (5)	C41—C46—C47—N11	0.1 (4)
C5—C6—C7—C8	−176.7 (4)	C47—N12—C48—N13	−1.9 (6)
C3—C2—C7—C6	−1.0 (6)	C47—N12—C48—C49	178.3 (3)
C1—C2—C7—C6	−179.3 (3)	C55—N13—C48—N12	178.5 (4)
C3—C2—C7—C8	177.8 (3)	Zn2—N13—C48—N12	−11.5 (5)
C1—C2—C7—C8	−0.5 (4)	C55—N13—C48—C49	−1.6 (4)
C9—N3—C8—N2	2.4 (6)	Zn2—N13—C48—C49	168.4 (2)
C9—N3—C8—C7	−178.7 (3)	N12—C48—C49—C50	0.1 (6)
C1—N2—C8—N3	179.8 (3)	N13—C48—C49—C50	−179.9 (4)
Zn1—N2—C8—N3	13.4 (5)	N12—C48—C49—C54	−179.3 (3)
C1—N2—C8—C7	0.8 (4)	N13—C48—C49—C54	0.8 (4)
Zn1—N2—C8—C7	−165.6 (2)	C54—C49—C50—C51	−1.6 (5)
C6—C7—C8—N3	−0.6 (6)	C48—C49—C50—C51	179.2 (4)
C2—C7—C8—N3	−179.2 (3)	C49—C50—C51—C52	0.1 (5)
C6—C7—C8—N2	178.5 (4)	C50—C51—C52—C53	1.5 (6)
C2—C7—C8—N2	−0.2 (4)	C51—C52—C53—C54	−1.7 (5)
C8—N3—C9—N4	−2.3 (6)	C52—C53—C54—C49	0.3 (5)
C8—N3—C9—C10	178.7 (3)	C52—C53—C54—C55	−179.0 (4)
C16—N4—C9—N3	179.8 (4)	C50—C49—C54—C53	1.4 (5)
Zn1—N4—C9—N3	−13.7 (5)	C48—C49—C54—C53	−179.2 (3)
C16—N4—C9—C10	−1.1 (4)	C50—C49—C54—C55	−179.2 (3)
Zn1—N4—C9—C10	165.5 (2)	C48—C49—C54—C55	0.2 (4)
N3—C9—C10—C11	1.4 (6)	C56—N14—C55—N13	2.4 (6)
N4—C9—C10—C11	−177.8 (4)	C56—N14—C55—C54	−176.8 (3)
N3—C9—C10—C15	179.8 (3)	C48—N13—C55—N14	−177.6 (3)
N4—C9—C10—C15	0.6 (4)	Zn2—N13—C55—N14	12.3 (5)
C15—C10—C11—C12	−1.2 (5)	C48—N13—C55—C54	1.8 (4)
C9—C10—C11—C12	177.1 (4)	Zn2—N13—C55—C54	−168.3 (2)
C10—C11—C12—C13	1.5 (6)	C53—C54—C55—N14	−2.5 (6)
C11—C12—C13—C14	−0.3 (6)	C49—C54—C55—N14	178.2 (3)
C12—C13—C14—C15	−1.2 (6)	C53—C54—C55—N13	178.1 (4)
C11—C10—C15—C14	−0.4 (6)	C49—C54—C55—N13	−1.2 (4)
C9—C10—C15—C14	−179.0 (3)	C55—N14—C56—N15	−2.1 (6)
C11—C10—C15—C16	178.7 (3)	C55—N14—C56—C57	177.5 (3)
C9—C10—C15—C16	0.0 (4)	C63—N15—C56—N14	178.8 (3)
C13—C14—C15—C10	1.6 (5)	Zn2—N15—C56—N14	−13.0 (5)
C13—C14—C15—C16	−177.2 (4)	C63—N15—C56—C57	−0.8 (4)
C17—N5—C16—N4	1.7 (6)	Zn2—N15—C56—C57	167.4 (2)
C17—N5—C16—C15	−178.6 (3)	N14—C56—C57—C58	0.3 (6)
C9—N4—C16—N5	−179.1 (4)	N15—C56—C57—C58	179.9 (4)
Zn1—N4—C16—N5	14.4 (5)	N14—C56—C57—C62	−179.8 (3)
C9—N4—C16—C15	1.1 (4)	N15—C56—C57—C62	−0.2 (4)
Zn1—N4—C16—C15	−165.4 (2)	C62—C57—C58—C59	−1.7 (5)
C10—C15—C16—N5	179.5 (3)	C56—C57—C58—C59	178.2 (4)
C14—C15—C16—N5	−1.6 (6)	C57—C58—C59—C60	0.4 (6)
C10—C15—C16—N4	−0.7 (4)	C58—C59—C60—C61	0.9 (6)
C14—C15—C16—N4	178.3 (4)	C59—C60—C61—C62	−1.0 (6)
C16—N5—C17—N6	−2.2 (6)	C60—C61—C62—C57	−0.3 (6)
C16—N5—C17—C18	174.6 (3)	C60—C61—C62—C63	−179.3 (4)
C24—N6—C17—N5	175.5 (4)	C58—C57—C62—C61	1.7 (6)
Zn1—N6—C17—N5	−13.6 (5)	C56—C57—C62—C61	−178.3 (3)
C24—N6—C17—C18	−1.6 (4)	C58—C57—C62—C63	−179.1 (3)
Zn1—N6—C17—C18	169.2 (2)	C56—C57—C62—C63	1.0 (4)
N5—C17—C18—C19	3.7 (7)	C64—N16—C63—N15	2.7 (6)
N6—C17—C18—C19	−179.0 (4)	C64—N16—C63—C62	−177.3 (4)
N5—C17—C18—C23	−175.8 (3)	C56—N15—C63—N16	−178.5 (4)
N6—C17—C18—C23	1.5 (4)	Zn2—N15—C63—N16	13.4 (5)
C23—C18—C19—C20	0.4 (6)	C56—N15—C63—C62	1.4 (4)
C17—C18—C19—C20	−179.0 (4)	Zn2—N15—C63—C62	−166.7 (2)
C18—C19—C20—C21	0.2 (6)	C61—C62—C63—N16	−2.4 (7)
C19—C20—C21—C22	−0.7 (6)	C57—C62—C63—N16	178.4 (3)
C20—C21—C22—C23	0.5 (6)	C61—C62—C63—N15	177.6 (4)
C21—C22—C23—C18	0.1 (6)	C57—C62—C63—N15	−1.5 (4)
C21—C22—C23—C24	179.7 (4)	C63—N16—C64—N17	−4.1 (6)
C19—C18—C23—C22	−0.6 (6)	C63—N16—C64—C65	177.1 (4)
C17—C18—C23—C22	179.0 (3)	C71—N17—C64—N16	−179.8 (4)
C19—C18—C23—C24	179.7 (3)	Zn2—N17—C64—N16	−10.6 (6)
C17—C18—C23—C24	−0.8 (4)	C71—N17—C64—C65	−0.9 (4)
C25—N7—C24—N6	2.1 (6)	Zn2—N17—C64—C65	168.2 (2)
C25—N7—C24—C23	−177.6 (3)	N16—C64—C65—C66	−0.9 (7)
C17—N6—C24—N7	−178.5 (4)	N17—C64—C65—C66	−179.8 (4)
Zn1—N6—C24—N7	10.5 (5)	N16—C64—C65—C70	179.7 (3)
C17—N6—C24—C23	1.2 (4)	N17—C64—C65—C70	0.8 (4)
Zn1—N6—C24—C23	−169.9 (2)	C70—C65—C66—C67	0.3 (6)
C22—C23—C24—N7	−0.2 (6)	C64—C65—C66—C67	−179.1 (4)
C18—C23—C24—N7	179.5 (3)	C65—C66—C67—C68	−0.4 (6)
C22—C23—C24—N6	−179.9 (4)	C66—C67—C68—C69	0.7 (6)
C18—C23—C24—N6	−0.2 (4)	C67—C68—C69—C70	−0.8 (6)
C24—N7—C25—N8	−0.3 (6)	C68—C69—C70—C65	0.6 (6)
C24—N7—C25—C26	179.1 (3)	C68—C69—C70—C71	179.9 (4)
C32—N8—C25—N7	179.0 (4)	C66—C65—C70—C69	−0.4 (6)
Zn1—N8—C25—N7	−13.6 (5)	C64—C65—C70—C69	179.1 (3)
C32—N8—C25—C26	−0.5 (4)	C66—C65—C70—C71	−179.8 (3)
Zn1—N8—C25—C26	166.9 (2)	C64—C65—C70—C71	−0.4 (4)
N7—C25—C26—C27	1.6 (6)	C40—N10—C71—N17	3.2 (6)
N8—C25—C26—C27	−178.9 (4)	C40—N10—C71—C70	−176.5 (3)
N7—C25—C26—C31	−179.8 (3)	C64—N17—C71—N10	−179.0 (4)
N8—C25—C26—C31	−0.3 (4)	Zn2—N17—C71—N10	11.8 (5)
C31—C26—C27—C28	0.7 (5)	C64—N17—C71—C70	0.7 (4)
C25—C26—C27—C28	179.1 (4)	Zn2—N17—C71—C70	−168.5 (2)
C26—C27—C28—C29	−0.7 (5)	C69—C70—C71—N10	0.2 (6)
C27—C28—C29—C30	0.3 (6)	C65—C70—C71—N10	179.5 (3)
C28—C29—C30—C31	0.1 (5)	C69—C70—C71—N17	−179.5 (4)
C29—C30—C31—C26	−0.2 (5)	C65—C70—C71—N17	−0.2 (4)
C29—C30—C31—C32	180.0 (4)	Zn2—N18—C72—C73	176.3 (3)
C27—C26—C31—C30	−0.2 (5)	N18—C72—C73—C78	1.9 (7)
C25—C26—C31—C30	−179.0 (3)	N18—C72—C73—C74	−178.8 (4)
C27—C26—C31—C32	179.6 (3)	C78—C73—C74—C75	3.8 (7)
C25—C26—C31—C32	0.9 (4)	C72—C73—C74—C75	−175.6 (4)
C1—N1—C32—N8	1.7 (6)	C73—C74—C75—C76	−1.6 (8)
C1—N1—C32—C31	−176.8 (3)	C74—C75—C76—C77	−1.7 (8)
C25—N8—C32—N1	−177.6 (3)	C75—C76—C77—C78	2.7 (8)
Zn1—N8—C32—N1	15.0 (5)	C74—C73—C78—C77	−2.8 (8)
C25—N8—C32—C31	1.1 (4)	C72—C73—C78—C77	176.6 (5)
Zn1—N8—C32—C31	−166.3 (2)	C76—C77—C78—C73	−0.5 (9)
C30—C31—C32—N1	−2.6 (6)	N19—C79—C80—C81	−147.6 (3)
C26—C31—C32—N1	177.5 (3)	N19—C79—C80—C85	28.2 (5)
C30—C31—C32—N8	178.6 (4)	C85—C80—C81—C82	0.0
C26—C31—C32—N8	−1.2 (4)	C79—C80—C81—C82	175.9 (3)
Zn1—N9—C33—C34	−178.2 (3)	C80—C81—C82—C83	0.0
N9—C33—C34—C35	−153.6 (4)	C81—C82—C83—C84	0.0
N9—C33—C34—C39	26.7 (7)	C82—C83—C84—C85	0.0
C39—C34—C35—C36	0.1 (7)	C83—C84—C85—C80	0.0
C33—C34—C35—C36	−179.7 (5)	C81—C80—C85—C84	0.0
C34—C35—C36—C37	−0.4 (7)	C79—C80—C85—C84	−175.8 (3)
C35—C36—C37—C38	0.1 (8)	N20—C86—C87—C88	−140.2 (9)
C36—C37—C38—C39	0.6 (7)	N20—C86—C87—C92	35.1 (14)
C37—C38—C39—C34	−0.9 (7)	C92—C87—C88—C89	0.0
C35—C34—C39—C38	0.6 (7)	C86—C87—C88—C89	176.0 (6)
C33—C34—C39—C38	−179.7 (5)	C87—C88—C89—C90	0.0
C71—N10—C40—N11	−3.4 (6)	C88—C89—C90—C91	0.0
C71—N10—C40—C41	174.8 (3)	C89—C90—C91—C92	0.0
C47—N11—C40—N10	176.6 (4)	C90—C91—C92—C87	0.0
Zn2—N11—C40—N10	−11.3 (5)	C88—C87—C92—C91	0.0
C47—N11—C40—C41	−1.8 (4)	C86—C87—C92—C91	−174.9 (8)
Zn2—N11—C40—C41	170.3 (2)	N21—C93—C94—C95	154.2 (6)
N10—C40—C41—C42	0.6 (6)	N21—C93—C94—C99	−28.7 (10)
N11—C40—C41—C42	179.1 (4)	C99—C94—C95—C96	0.0
N10—C40—C41—C46	−176.6 (3)	C93—C94—C95—C96	177.3 (6)
N11—C40—C41—C46	1.9 (4)	C94—C95—C96—C97	0.0
C46—C41—C42—C43	1.0 (6)	C95—C96—C97—C98	0.0
C40—C41—C42—C43	−175.9 (4)	C96—C97—C98—C99	0.0
C41—C42—C43—C44	−0.7 (6)	C97—C98—C99—C94	0.0
C42—C43—C44—C45	0.2 (6)	C95—C94—C99—C98	0.0
C43—C44—C45—C46	0.2 (6)	C93—C94—C99—C98	−176.9 (6)

### Hydrogen-bond geometry (Å, º)

Cg1 and Cg2 are the centroids of the C94–C99 and N4/C9/C10/C15/C16 rings, respectively.

**Table:**

*D*—H···*A*	*D*—H	H···*A*	*D*···*A*	*D*—H···*A*
N9—H9*B*···N19	0.88	2.22	3.093 (5)	171
N18—H18*A*···N20	0.88	2.29	3.082 (6)	150
N20—H20*A*···N16	0.88	2.52	3.277 (6)	145
N21—H21*B*···N12	0.88	2.45	3.320 (9)	169
N9—H9*A*···*Cg*1^i^	0.88	2.85	3.710 (4)	165
N19—H19*A*···*Cg*2	0.88	2.91	3.407 (4)	117

Symmetry code: (i) −*x*+1/2, −*y*+1, *z*+1/2.
